# One-Pot Synthesis of Novel Multisubstituted 1-Alkoxyindoles

**DOI:** 10.3390/molecules26051466

**Published:** 2021-03-08

**Authors:** Ye Eun Kim, Hyunsung Cho, Yoo Jin Lim, Chorong Kim, Sang Hyup Lee

**Affiliations:** College of Pharmacy and Innovative Drug Center, Duksung Women’s University, Seoul 01369, Korea; kimye92@duksung.ac.kr (Y.E.K.); sungcho2013@duksung.ac.kr (H.C.); esther1109@duksung.ac.kr (Y.J.L.); giliiin0818@duksung.ac.kr (C.K.)

**Keywords:** 1-alkoxyindoles, stannous chloride, nitro reduction, intramolecular cyclization, nitrone, *O*-alkylation

## Abstract

Studies on a one-pot synthesis of novel multisubstituted 1-alkoxyindoles **1** and their mechanistic investigations are presented. The synthesis of **1** was successfully achieved through consecutive four step reactions from substrates **2**. The substrates **2**, prepared through a two-step synthetic sequence, underwent three consecutive reactions of nitro reduction, intramolecular condensation, and nucleophilic 1,5-addition to provide the intermediates, 1-hydroxyindoles **8**, which then were alkylated in situ with alkyl halide to afford the novel target products **1**. We optimized the reaction conditions for **1** focusing on the alkylation step, along with the consideration of formation of intermediates **8**. The optimized condition was SnCl_2_·2H_2_O (3.3 eq) and alcohols (R^1^OH, 2.0 eq) for 1–2 h at 40 °C and then, base (10 eq) and alkyl halides (R^2^Y, 2.0 eq) for 1–4 h at 25–50 °C. Notably, all four step reactions were performed in one-pot to give **1** in good to modest yields. Furthermore, the mechanistic aspects were also discussed regarding the reaction pathways and the formation of side products. The significance lies in development of efficient one-pot reactions and in generation of new 1-alkoxyindoles.

## 1. Introduction

**Figure 1:**
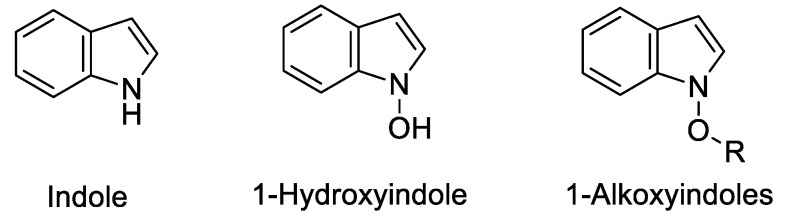
Indole, 1-hydroxyindole, and 1-alkoxyindoles.

1-Alkoxyindoles are the compounds that are similar to the indole structure, but have an alkoxy group (-OR) instead of H at the N(1) site, as shown in Figure 1. Due to the presence of alkoxy group, 1-alkoxyindole compounds are supposed to have different physical and chemical properties compared to indole compounds. While an indole structure is commonly found in natural products, the 1-alkoxyindole structure rarely appears. Recently, 1-alkoxyindole derivatives have been emerging as alternative compounds to indole compounds. Due to the 1-hydroxy group, 1-hydroxyindoles are known to be more polar and acidic (*p*K_a_ 8.1–9.8) than indoles, leading to higher exposure of the polar 1-OH group and easier deprotonation followed by further functionalization [1]. Natural products and synthetic derivatives including the 1-hydroxyindole and 1-alkoxyindole structure have attracted much attention due to their various biological activities. For example, stephacidin B [2], tetrahydro-β-carboline derivatives [3], and (*R*)-paniculidine B [4] have cytotoxic activities and, in particular, nocathiacin is known as a promising natural antibiotic [5]. Synthetic 1-hydroxyindole compounds have also shown inhibitory activities for lactate dehydrogenase-A (LDH-A) [6]. *N*-alkoxyindole-3-carbinol (I3C) compounds are proved to regulate cell cycle-related gene transcription and as a result exhibit inhibitory activities in human breast cancer cell lines [7]. Despite these biological activities of natural and synthetic products containing 1-hydroxyindole and 1-alkoxyindole structures, their derivatizations have not been extensively studied due to the lack of tolerable synthetic methods and the instability of those compounds.

Since the hydroxy group in 1-hydroxyindole compounds is a kind of nucleophile, alkylations on that site could occur smoothly in the presence of bases. Thus, 1-methoxyindoles, a typical class of 1-alkoxyindoles, could be synthesized through the methylation of 1-hydroxyindole using methyl iodide, diazomethane, or dimethyl sulfate in the presence of an appropriate base [1,8]. As precursors of 1-alkoxyindoles, 1-hydroxyindoles have also drawn our attention. Previously, the 1-hydroxyindole derivative was successfully applied for the total synthesis of nocathiacin [9]. We also reported methods to synthesize 2,3-disubstituted 1-hydroxyindole compounds [10,11,12,13]. Other methods by reduction of the indole system to 2,3-dihydroindole, followed by an oxidation (Na_2_WO_4_/H_2_O_2_) were applied to give 1-hydroxyindoles [14]. The other methods include the direct synthesis of 1-alkoxyindoles through intramolecular cyclization of the alkoxyimine structure, without using 1-hydroxyindole as an intermediate [15]. However, these methods have suffered from a narrow range of derivative diversity, low yields, and poor reproducibility due to their chemical instabilities.

**Figure 2:**
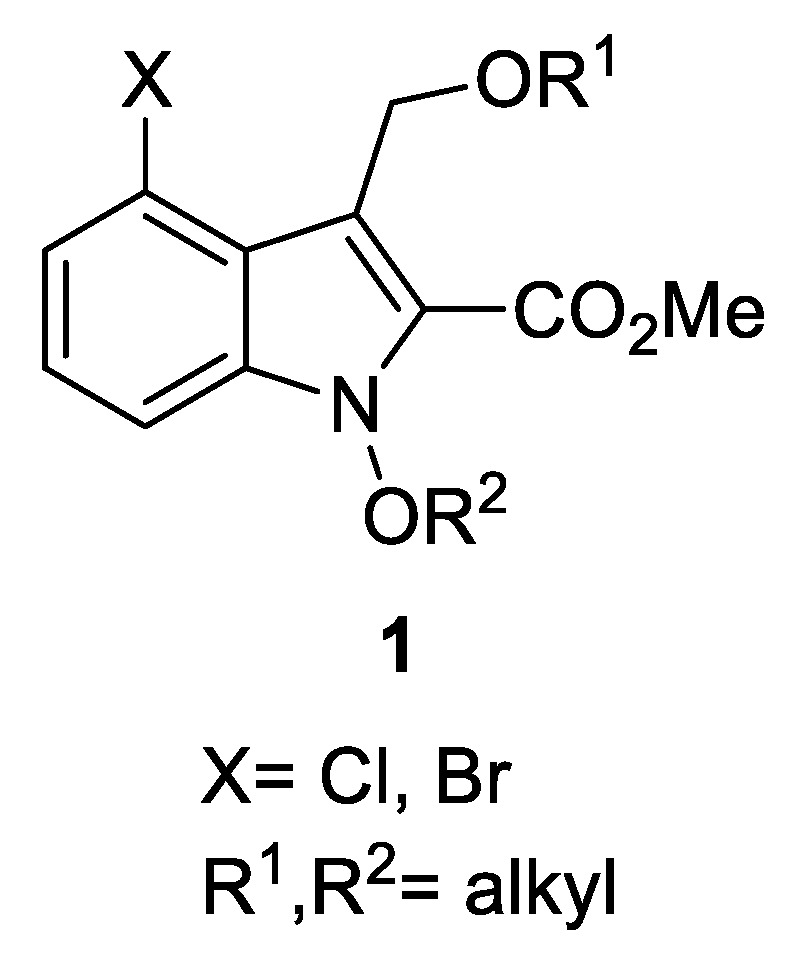
Structures of multisubstituted 1-alkoxyindoles **1**.

For decades, chemical instabilities have hampered the extensive studies on these compounds. So, we aimed to design and synthesize the compounds of improved chemical stability compared to 1-hydroxyindoles, with developing tolerable synthetic methods. It is believed that an alkyl or acyl group directly connected to the indole skeleton could stabilize the 1-hydroxyindole structure. For an example, 1-alkylation of the 1-hydroxy group is believed to improve the stability of those compounds [16]. Thus, we focus on creating new derivatives of multisubstituted alkoxyindole by introducing various groups at C(2), C(3), and C(4), and alkyl groups at N(1) in 1-hydroxyindoles, expecting to improve their chemical stabilities, as shown in Figure 2. It is also meaningful in medicinal chemistry to further expect the improved absorption in the body by lowering the polarity of the compounds with the alkylation. The significance of our study lies in the development of efficient one-pot reactions of a four-step sequence, and in creation of new 1-alkoxyindole compounds with improved stabilities, otherwise difficult to synthesize.

## 2. Results and Discussion

### 2.1. Synthesis of Conjugate Nitro Ketoesters

**Scheme 1:**
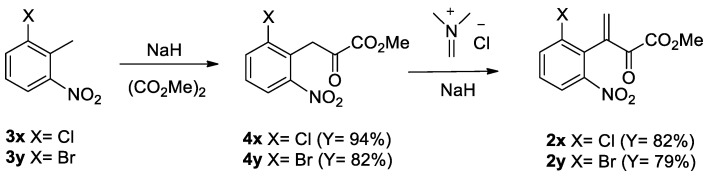
Synthesis of conjugate nitro ketoesters **2**.

First, we needed to prepare the required substrates to synthesize the target compounds **1**. For the synthesis of **2** we applied our previous two-step synthetic sequence [17,18,19] with minor modifications, as shown in Scheme 1. Nitrotoluenes **3** were reacted with sodium hydride and dimethyl oxalate in the *N,N*-dimethylformamide (DMF) solvent to afford ketoesters **4** in excellent yields. In this process, excess sodium hydride and efficient degassing processes were applied to produce **4** in improved yields. Then, ketoesters **4** were treated with sodium hydride and dimethylmethyleneiminium chloride in tetrahydrofuran (THF) to afford conjugate nitro ketoesters **2** in good yields. As a result, the synthesis of substrates **2** were achieved in improved yields compared with previous results [19].

### 2.2. Optimization for Formation of 1-Alkoxyindoles ***1***

**Scheme 2:**
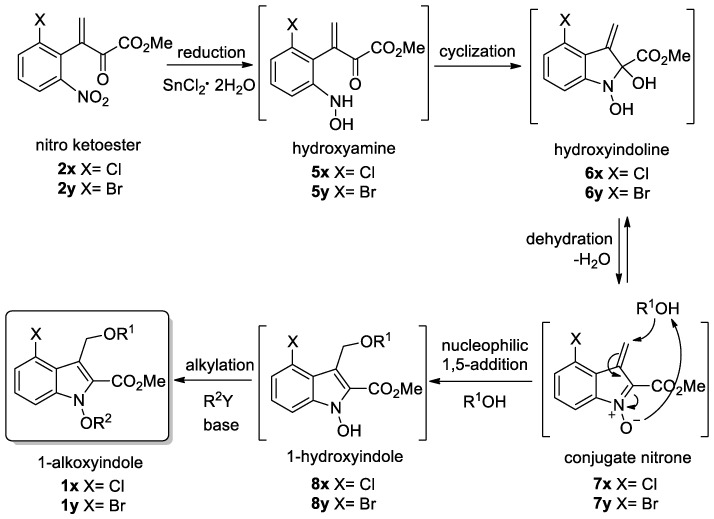
Synthetic pathway for multisubstituted 1-alkoxyindoles **1**.

We adopted SnCl_2_·2H_2_O as a reducing agent for our reactions according to our previous procedures for intermediates **8** [9,17]. As shown in Scheme 2, substrates **2** undergo a reduction by SnCl_2_·2H_2_O to give hydroxyamines **5**, intramolecular cyclization (addition) to give hydroxyindolines **6**, dehydration to give conjugate nitrones **7**, and 1,5-addition of nucleophile (R^1^OH) to afford the intermediate 1-hydroxyindoles **8** and then, **8** undergo alkylation with alkyl halides (R^2^Y) to provide the target compounds, 1-alkoxyindoles **1**, which were all run in one-pot.

We then tried to optimize the reaction conditions for target compounds **1** including the intermediates **8**. Although the procedures for intermediates **8** were already known [9,17], we had to reoptimize the whole processes since the following alkylation reaction to form **1** could be inevitably affected by the condition for intermediates **8**. For examples, the remained nucleophile (R^1^OH) could interfere with the following alkylation step by reacting with alkyl halide (R^2^Y), and the excess amount of SnCl_2_·2H_2_O could also affect the alkylation step. Furthermore, the selection of the appropriate base for the alkylation step would be important. Considering these, we first tried to conduct the reactions with several bases in the preliminary-optimized condition (2 h, 40 °C for **8y**; 2 h, 25 °C for **1y**). So, we tested triethylamine (TEA), *N,N*-diisopropylethylamine (DIEA), 4-dimethylaminopyridine (DMAP), and 1,8-diazabicyclo[5,4,0]undec-7-ene (DBU) as organic bases, and K_2_CO_3_ as an inorganic base. With **2y** and SnCl_2_·2H_2_O (3.3 eq), we chose benzyl alcohol (BnOH, 2 eq) as a template nucleophile and benzyl bromide (BnBr, 2 eq) as a template alkyl halide with base (10 eq) affording 1-benzyloxyindole **1yh**, and the results were shown in Table 1. Among organic bases, DBU provided the best yield (41%), followed by TEA (23%), DIEA (17%), and DMAP (13%). The inorganic base K_2_CO_3_ gave poor yield. The order of basicity of organic bases (DMAP < TEA < DIEA < DBU) [20,21] roughly corresponded with the order of yields, except TEA and DIEA cases. The nucleophilicity of 1-OH could be activated by base and so a stronger base could afford better yield. Accordingly, the DBU (a guanidine-type base) gave the best result. When we varied the amount of bases, the results were not improved much. Thus, we chose DBU for our further reactions.

With the selected base DBU and substrate **2x**, we first attempted to perform the systematic studies on the reaction conditions suitable for formation of 1-benzyloxyindole **1xh**. As the whole sequence of reactions could be triggered by reduction of the aromatic nitro group, the amount of the reducing agent would be considered one of the most important factors. Thus, we optimized the reaction conditions by varying the amount of SnCl_2_·2H_2_O (2.5–3.7 eq). We also varied the amount of nucleophile (BnOH), base (DBU) and alkyl halide (R^2^Y), and the results were shown in Table 2. Notably, since the reducing agent SnCl_2_·2H_2_O could be hydrolyzed to give HCl that makes the reaction media acidic, we used a large amount of base (DBU) in proportion to the amount of SnCl_2_·2H_2_O. We first tested the amount of SnCl_2_·2H_2_O (2.5–3.7 eq) and, correspondingly, DBU (7.6–11.2 eq). In general, lower levels and higher level of those reagents than 3.3 eq for SnCl_2_·2H_2_O and 10 eq for DBU, respectively, provided poor yields (entries 1, 2, and 9, Table 2). So, under this condition we further tested the amount of nucleophile (BnOH) and alkyl halide (BnBr). In general, higher amounts of nucleophile and alkyl halide than 2.0 eq provided higher yields (32 → 33 → 42 → 45%) (entries 5–8, Table 2). However, one issue was involved in those reactions with higher amounts of them. Excess alcohols in 1,5-addition reaction could react with alkyl halide in the alkylation step, producing dialkyl ethers. As expected, we observed the formation of large amount of dibenzyl ether as a byproduct, which is difficult to remove. In addition to the isolation problem, the economical reaction efficiencies were poor due to the excess amount of reagents and the large amount of byproducts. Considering these points, we adopted the condition of 2 eq of nucleophile and alkyl halide (entry 5, Table 2) despite the slightly lower yield. Taken together, we chose the optimized conditions for **1**; 1.0 eq of **2x**, 3.3 eq of SnCl_2_·2H_2_O, 2.0 eq of BnOH (2 h, 40 °C), and then 10.0 eq of DBU, 2 eq of BnBr (2 h, 25 °C), which was applied to all other reactions, unless otherwise noted. Notably, in this method, 1-alkoxyindoles **1** were synthesized in one-pot without separation of the intermediates **8**. We compared the result of reactions with isolation and without isolation of the intermediate **8xh**, and found that the yield (32%) of **1xh** in the one-pot reaction was higher than that (28%) of **1xh** in two separate reactions (45% for **8xh** and 62% for **1xh**).

### 2.3. Synthesis of New Derivatives of 1-Alkoxyindoles ***1***

Using the optimized condition, various new derivatives of **1** were synthesized, as shown in Table 3. The substrates **2** were treated with SnCl_2_·2H_2_O in dimethoxyethane (DME) for 1–2 h at 40 °C in the presence of a nucleophile and 4Å molecular sieves to give the intermediates **8**, which were then alkylated in situ with alkyl halide in the presence of DBU for 1–4 h at 25–50 °C in one-pot, finally affording to the target compounds **1**. In the alkylation step, vigorous stirring was required to make the reaction mixture a good suspension condition. Here, we used various alcohols as nucleophiles to synthesize **1xa**-**1ym** (22 new derivatives). When primary alcohols and primary alkyl halides were used (entries 1–9, 12–19, and 22, Table 3), the reactions provided **1** in fairly good yields for four-step sequence (18–52%). Interestingly, when we compared the results of **1x** series by the size of alkyl groups in both alcohols and alkyl halides, we found that the reaction using methanol with methyl iodide (methyl–methyl case, **1xa**) gave the best yield (43%). The reaction using methanol with octyl bromide (methyl–octyl case, **1xm**) gave 33% yield, and the reaction using octanol with methyl iodide (octyl–methyl case, **1xn**) gave a similar yield (36%). The reactions for ethyl–ethyl (**1xb**), propyl–propyl (**1xc**), and butyl–butyl (**1xd**) cases gave modest yields (30–32%). The reactions for pentyl–pentyl (**1xe**), hexyl–hexyl (**1xf**), and octyl–octyl (**1xg**) cases gave relatively poor yields (20–22%). These observations implicated that the size of alkyl groups seems to affect the results, providing better yields with smaller alkyl groups, and that both nucleophilic addition of alcohol and alkylation of 1-OH group seem to influence the final results. Reactions with secondary alcohols and secondary alkyl halides (entries 10, 11, and 20, Table 3) gave **1** in relatively low yields (11–16%). The reaction with cyclohexyl bromide did not successfully proceed at 25 °C, so we elevated reaction temperature to 50 °C for an extended time (4 h), obtaining acceptable yield (11%, entry 11, Table 3). This implied that the steric effect of alkyl halides might have an influence on alkylation of 1-hydroxyindoles. Notably, the reactions with the methyl group (entries 1 and 15) and benzyl group (entries 8 and 19) afforded higher yields than the other cases. In addition, comparing the reactions for **1x** (Cl group) and **1y** (Br group), we found no consistent trends despite slightly higher yields for **1y** in some cases. 

### 2.4. Mechanistic Investigations on Reaction Pathways

**Scheme 3:**
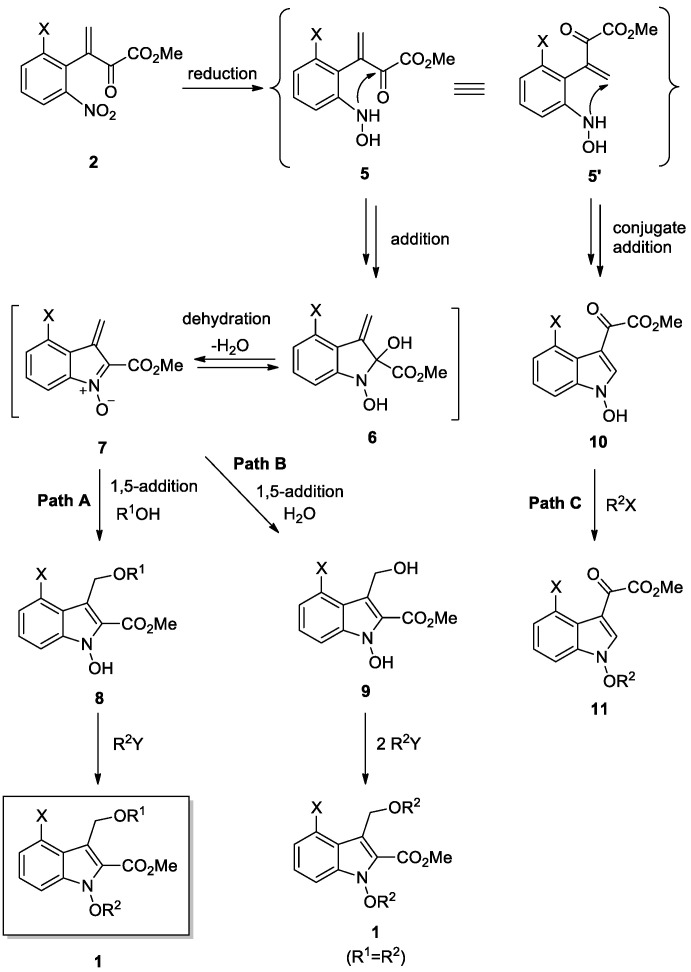
Proposed pathways for **1** and **11**.

We investigated the reaction mechanisms and pathways based on the generated products, as shown in Scheme 3. Reduction of the substrates **2** could give two conformers of hydroxyamine, **5** and **5’**. Here are three following pathways involved, pathways A, B, and C. Pathways A and B proceed through conformer **5**, and pathway C through conformer **5’**. We first investigated the reactions through conformer **5,** which then could undergo intramolecular condensation (addition and dehydration) to give conjugate nitrone **7**. Subsequent 1,5-addition of **7** with a nucleophile (R^1^OH) would give 1-hydroxyindoles **8,** which then undergo alkylation with alkyl halide (R^2^Y) to give 1-alkoxyindoles **1** (Path A). Interestingly, in the process of 1,5-nucleophilic addition of **7**, H_2_O could react as a nucleophile instead of alcohol, leading to the generation of dihydroxy species **9** (Path B). This species **9** could also react with 2.0 eq of alkyl halide (R^2^Y) to produce 1-alkoxindoles **1** (R^1^ = R^2^). Furthermore, the conformer **5’** could undergo intramolecular conjugate addition to give another type of 1-hydroxyindole compounds **10** with a different skeleton, which is consistent with the previous observation [9]. These compounds **10** would also undergo alkylation with alkyl halide to give 1-alkoxyindole-3-carboxylates **11** that are considered as rearranged products compared to **1**. However, the formation of compounds **10** and **11** was variable and, sometimes, it was difficult to isolate and identify these compounds. When we conducted the reactions for **1xl** and **1xm** (entries 12 and 13), we observed the formation **11xl** (R^2^ = Me, 32% yield) and **11xm** (R^2^ = Oct, 3% yield) along with the main products **1xl** and **1xm**, respectively. In most of the reactions in Table 3, a substantial amount (10–35%) of rearranged products **11** were also formed, which might explain the low yields of the products **1**. In addition, despite full conversion of the starting material in the reactions, significant tarring and side products might cause low yields.

## 3. Materials and Methods

### 3.1. General Methods

Reagents used in this study were purchased from Sigma-Aldrich (Darmstadt, Germany), Thermo Fisher (Waltham, MA, USA) and TCI (Tokyo, Japan). They were of commercial quality and used without further purification, unless otherwise stated. Reactions were periodically monitored by thin-layer chromatography (TLC) carried on 0.25 mm Merck silica gel plates (20 cm × 20 cm; Merck F254) (Darmstadt, Germany) and visualized under UV light. Purifications were performed by preparative TLC (PTLC) and column chromatography. PTLC separations were carried out on the same silica gel plates. Column chromatography was performed using Merck silica gels (230–400 mesh) (Zvornik, Bosnia and Herzegovina). Melting points were determined in Deckgläser Cover Glasses (Lauda-Königshofen, Germany) using a Thermo Scientific 00590Q apparatus (Dubuque, Iowa, USA). ^1^H (300 MHz) and ^13^C (75 MHz) NMR spectra were recorded on a Bruker DRX 300 spectrometer (Zürich, Switzerland) (See Supplementary Materials) and chemical shifts (δ) are expressed relative to tetramethylsilane (TMS). Mass spectra were obtained in EI or ESI ionization modes (Agilent, Santa Clara, CA, USA). High resolution mass spectra were obtained using JEOL apparatus (Tokyo, Japan) at the Korea Basic Science Institute, Republic of Korea. HPLC analyses were performed using the following Waters Associate Units: 515 A pump, 515 B pump, dual λ absorbance 2487 detector, 717 plus autosampler, and COSMOSIL 5C_18_-AR-Ⅱ Packed Column (4.6 mm × 250 mm) (Worcester, MA, USA). The product analyses were performed using a linear gradient condition: from 70% A (aqueous) and 30% B (acetonitrile) for 3 min (isocratic), then to 10% A and 90% B in 30 min (gradient). Finally, keep 10% A and 90% B for 5 min. The flow rate was 1 mL/min with eluent monitoring at 254 nm. HPLC solvents were filtered (aqueous solution with Millipore HVLP, 0.45 mm; MeCN with Millipore HV, 0.45 mm) and degassed before use.

### 3.2. Substrate Synthesis

*Methyl 3-(2′-Chloro-6′-nitrophenyl)-2-oxopropanoate (* **4x** *) [19]*

Dimethyl oxalate (4.02 g, 34.0 mmol, 5.0 eq) and 2-chloro-6-nitrotoluene (**3x**, 1.17 g, 6.8 mmol, 1.0 eq) was dissolved in anhydrous DMF (8.2 mL). To a stirred mixture of NaH (60% in mineral oil, 1.09 g, 27.2 mmol, 4.0 eq) in anhydrous DMF (4.1 mL) at 0 °C was added dropwise a solution of dimethyl oxalate and 2-chloro-6-nitrotoluene. Stirring was continued for 1 h at 0 °C during which it turned a reddish-brown. The reaction mixture was allowed to warm to 25 °C and stirred for an additional 4 h. The reaction mixture turned dark red. The reaction mixture was quenched with saturated NH_4_Cl (15 mL) at 0 °C, extracted with EtOAc (2 × 20 mL) and washed with H_2_O (2 × 20 mL). The organic layer was dried (MgSO_4_) and concentrated in vacuo. The residue was purified by column chromatography (1:4 → 1:2 EtOAc/hexanes) to get the compound **4x** (1.65 g, 94%) as a pale-yellow solid. Mp 58–59 °C; *R*_f_ 0.24 (1:4 EtOAc/hexanes); HPLC t*_R_*21.2 min; ^1^H NMR (300 MHz, CDCl_3_): δ 8.00 (dd, *J* = 8.2, 1.1 Hz, 1H, Ar), 7.74 (dd, *J* = 8.2, 1.1 Hz, 1H, Ar), 7.46 (t, *J* = 8.2 Hz, 1H, Ar), 4.72 (s, 2H, C(2)CH_2_), 3.96 (s, 3H, CO_2_CH_3_); ^13^C NMR (75 MHz, CDCl_3_): δ 188.0 (*C*OCO_2_CH_3_), 160.1 (*C*O_2_CH_3_), 150.5 (Ar), 137.7 (Ar), 134.7 (Ar), 129.0 (Ar), 127.6 (Ar), 124.0 (Ar), 53.8 (CO_2_*C*H_3_), 42.8 (C(2)*C*H_2_); MS *m*/*z* 257 [M]^+^; HRMS (+ESI) calcd for C_10_H_8_ClNNaO_5_ [M + Na]^+^ 279.9989, found 279.9983. Spectral data are in accordance with literature information [19].

*Methyl 3-(2′-Bromo-6′-nitrophenyl)-2-oxopropanoate (* **4y** *) [19]*

Dimethyl oxalate (2.01 g, 17.0 mmol, 5.0 eq) and 2-bromo-6-nitrotoluene (**3y**, 735 mg, 3.4 mmol, 1.0 eq) was dissolved in anhydrous DMF (4.1 mL). To a stirred mixture of NaH (60% in mineral oil, 544 mg, 13.6 mmol, 4.0 eq) in anhydrous DMF (2.04 mL) at 0 °C was added dropwise a solution of dimethyl oxalate and 2-bromo-6-nitrotoluene. Stirring was continued for 1 h at 0 °C during which it turned a reddish-brown. The reaction mixture was allowed to warm to 25 °C and stirred for additional 4 h. The reaction mixture turned dark red. The reaction mixture was quenched with saturated NH_4_Cl (10 mL) at 0 °C, extracted with EtOAc (2 × 10 mL), and washed with H_2_O (2 × 10 mL). The organic layer was dried (MgSO_4_) and concentrated in vacuo. The residue was purified by column chromatography (1:4 → 1:2 EtOAc/hexanes) to get the compound **4y** (848 mg, 82%) as a pale-yellow solid. Mp 70 °C; *R*_f_ 0.22 (1:4 EtOAc/hexanes); HPLC t*_R_* 21.7 min; ^1^H NMR (300 MHz, CDCl_3_): δ 8.02 (dd, *J* = 8.2, 1.1 Hz, 1H, Ar), 7.92 (dd, *J* = 8.2, 1.1 Hz, 1H, Ar), 7.39 (t, *J* = 8.2 Hz, 1H, Ar), 4.75 (s, 2H, C(2)CH_2_), 3.97 (s, 3H, CO_2_CH_3_); ^13^C NMR (75 MHz, CDCl_3_): δ 187.8 (*C*OCO_2_CH_3_), 160.7 (*C*O_2_CH_3_), 150.6 (Ar), 138.0 (Ar), 129.7 (Ar), 129.2 (Ar), 128.4 (Ar), 124.6 (Ar), 53.7 (CO_2_*C*H_3_), 43.9 (C(2)*C*H_2_); MS *m*/*z* 301 [M]^+^; HRMS (+ESI) calcd for C_10_H_8_BrNNaO_5_ [M + Na]^+^ 323.9484, found 323.9475. Spectral data are in accordance with literature information [19].

*Methyl 3-(2′-Chloro-6′-nitrophenyl)-2-oxobut-3-enoate (* **2x** *) [19]*

Ketoester (**4x**, 1.04 g, 4.03 mmol, 1.0 eq) was dissolved in anhydrous THF (34 mL). To a stirred mixture of NaH (60% in mineral oil, 178 mg, 4.43 mmol, 1.1 eq) in anhydrous THF (65 mL) at 0 °C was added dropwise a solution of ketoester. After stirring for 1 h at 0 °C, *N,N*-dimethylmethyleneiminium chloride (1.3 g, 12.08 mmol, 3.0 eq) was added and stirred for 1 h at 0 °C. The reaction mixture was allowed to warm to 25 °C and stirred for additional 3 h. The reaction mixture turned pale-yellow. The reaction mixture was quenched with saturated NH_4_Cl (10 mL) at 0 °C, extracted with EtOAc (2 × 50 mL), and washed with H_2_O (2 × 50 mL). The organic layer was dried (MgSO_4_) and concentrated in vacuo. The residue was purified by column chromatography (1:4 → 1:1 EtOAc/hexanes) to get the compound **2x** (890 mg, 82%) as a pale-yellow solid. Mp 67–68 °C; *R*_f_ 0.44 (1:2 EtOAc/hexanes); HPLC t*_R_* 21.7 min; ^1^H NMR (300 MHz, CDCl_3_): δ 7.99 (dd, *J* = 8.2, 1.1 Hz, 1H, Ar), 7.75 (dd, *J* = 8.2, 1.1 Hz, 1H, Ar), 7.50 (t, *J* = 8.2 Hz, 1H, Ar), 6.80 (s, 1H, C*H*H), 6.19 (s, 1H, CH*H*), 3.93 (s, 1H, OCH_3_); ^13^C NMR (75 MHz, CDCl_3_): δ 183.0 (*C*OCO_2_CH_3_), 162.5 (*C*O_2_CH_3_), 149.7 (C(2)*C*CH_2_), 139.8 (Ar), 136.3(C(2)C*C*H_2_), 134.7 (Ar), 134.5 (Ar), 130.6 (Ar), 130.1 (Ar), 123.2 (Ar), 53.3 (OCH_3_); MS *m*/*z* 269 [M]^+^; HRMS (+ESI) calcd for C_11_H_8_ClNNaO_5_ [M + Na]^+^ 291.9989, found 291.9983. Spectral data are in accordance with literature information [19].

*Methyl 3-(2′-Bromo-6′-nitrophenyl)-2-oxobut-3-enoate (* **2y** *) [19]*

Ketoester (**4y**, 829 mg, 2.74 mmol, 1.0 eq) was dissolved in anhydrous THF (23 mL). To a stirred mixture of NaH (60% in mineral oil, 121 mg, 3.02 mmol, 1.1 eq) in anhydrous THF (46 mL) at 0 °C was added dropwise a solution of ketoester. After stirring for 1 h at 0 °C, *N,N*-dimethylmethyleneiminium chloride (770 mg, 8.23 mmol, 3.0 eq) was added and stirred for 1 h at 0 °C. The reaction mixture was allowed to warm to 25 °C and stirred for additional 3 h. The reaction mixture turned pale yellow. The reaction mixture was quenched with saturated NH_4_Cl (10 mL) at 0 °C, extracted with EtOAc (2 × 50 mL) and washed with H_2_O (2 × 50 mL). The organic layer was dried (MgSO_4_) and concentrated in vacuo. The residue was purified by column chromatography (1:4 → 1:1 EtOAc/hexanes) to get the compound **2y** (680 mg, 79%) as a pale-yellow solid. Mp 80 °C; *R*_f_ 0.44 (1:2 EtOAc/hexanes); HPLC t*_R_* 21.9 min; ^1^H NMR (300 MHz, CDCl_3_): δ 8.02 (dd, *J* = 8.2, 1.1 Hz, 1H, Ar), 7.94 (dd, *J* = 8.2, 1.1 Hz, 1H, Ar), 7.44 (t, *J* = 8.2 Hz, 1H, Ar), 6.79 (s, 1H, C*H*H), 6.17 (s, 1H, CH*H*), 3.94 (s, 3H, OCH_3_); ^13^C NMR (75 MHz, CDCl_3_): δ 183.0 (*C*OCO_2_CH_3_), 162.5 (*C*O_2_CH_3_), 149.7 (C(2)*C*CH_2_), 141.7 (Ar), 137.8 (C(2)C*C*H_2_), 134.4 (Ar), 132.4 (Ar), 130.4 (Ar), 126.2 (Ar), 123.8 (Ar), 53.3 (OCH_3_); MS *m*/*z* 313 [M]^+^; HRMS (+ESI) calcd for C_11_H_8_BrNNaO_5_ [M + Na]^+^ 335.9484, found 335.9474. Spectral data are in accordance with literature information [19].

### 3.3. General Procedure for the Synthesis of 1-Alkoxyindoles ***1***

SnCl_2_·2H_2_O and 4Å molecular sieves stirred in DME for 30 min at 25 °C. To a stirred mixture was added alcohol and conjugate ketoester **2**. The resulting mixture was stirred for 1–2 h at 40 °C. After checking that the starting material was disappeared by using TLC, DBU was added and stirred strongly for 30 min at 25 °C. The alkyl halide was then added and stirring was continued for 1–4 h at 25–50 °C until reaction completed. The reaction mixture was diluted with CH_2_Cl_2_ and washed with brine. The organic layer was dried (MgSO_4_) and concentrated in vacuo to afford a crude residue. The residue was purified by preparative TLC (PTLC) and column chromatography to give 1-alkoxyindoles **1**. Spectral data of all compounds were in good accordance with the literature information.

*Methyl 4-Chloro-1-methoxy-3-(methoxymethyl)-1H-indole-2-carboxylate (* **1xa** *)*

Use of SnCl_2_·2H_2_O (82.8 mg, 0.37 mmol, 3.3 eq), methanol (9 μL, 0.22 mmol, 2.0 eq), and **2x** (30 mg, 0.11 mmol, 1.0 eq) for 1 h at 40 °C then use of DBU (165 μL, 1.10 mmol, 10.0 eq) and methyl iodide (14 μL, 0.22 mmol, 2.0 eq) for 1 h at 25 °C in general procedure afforded the title compound **1xa** (13.7 mg, 43%) as a pale-yellow solid. Mp 59–60 °C; *R*f 0.40 (1:4 EtOAc/hexanes); HPLC t*_R_* 20.8 min; UV vis (CH_3_CN-H_2_O) λ_max_ 236, 298 nm; ^1^H NMR (300 MHz, CDCl_3_): δ 7.38 (d, *J* = 8.2 Hz, 1H, Ar), 7.26 (t, *J* = 8.4 Hz, 1H, Ar), 7.18 (d, *J* = 5.9 Hz, 1H, Ar), 5.07 (s, 2H, C(3)CH_2_O), 4.18 (s, 3H, N(1)OCH_3_), 4.01 (s, 3H, CO_2_CH_3_), 3.45 (s, 3H, CH_2_OC*H*_3_); ^13^C NMR (75 MHz, CDCl_3_): δ 160.8 (C=O), 135.9 (Ar), 128.6 (Ar), 126.4 (Ar), 125.3 (Ar), 123.1 (Ar), 119.7 (Ar), 116.4 (Ar), 108.2 (Ar), 66.4 (N(1)OCH_3_), 63.7(CH_2_O*C*H_3_), 58.1 (C(3)*C*H_2_O), 52.4 (CO_2_*C*H_3_); MS *m*/*z* 283 [M]^+^; HRMS (+ESI) calcd for C_13_H_14_ClNNaO_4_ [M + Na]^+^ 306.0509, found 306.0509.

*Methyl 4-Chloro-1-ethoxy-3-(ethoxymethyl)-1H-indole-2-carboxylate (* **1xb** *)*

Use of SnCl_2_·2H_2_O (140 mg, 0.62 mmol, 3.3 eq), ethanol (22 μL, 0.37 mmol, 2.0 eq) and **2x** (50 mg, 0.185 mmol, 1.0 eq) for 1 h at 40 °C, then use of DBU (272 μL, 1.85 mmol, 10.0 eq) and bromoethane (28 *μ*L, 0.37 mmol, 2.0 eq) for 1 h at 25 °C in general procedure afforded the title compound **1xb** (17.8 mg, 31%) as a pale-yellow solid. Mp 42 °C; *R*_f_ 0.49 (1:4 EtOAc/hexanes); HPLC t*_R_* 25.8 min; UV vis (CH_3_CN-H_2_O) λ_max_ 235, 298nm; ^1^H NMR (300 MHz, CDCl_3_): δ 7.34 (d, *J* = 8.1 Hz, 1H, Ar), 7.23 (t, *J* = 7.8 Hz, 1H, Ar), 7.15 (d, *J* = 7.0 Hz, 1H, Ar), 5.09 (s, 2H, C(3)CH_2_O), 4.41 (q, *J* = 7.1 Hz, 2H, N(1)OCH_2_), 3.99 (s, 3H, CO_2_CH_3_), 3.65 (q, *J* = 7.0 Hz, 2H, OC*H*_2_CH_3_), 1.44 (t, *J* = 7.1 Hz, 3H, N(1)OCH_2_C*H*_3_), 1.25 (t, *J* = 7.0 Hz, 3H, OCH_2_C*H*_3_); ^13^C NMR (75 MHz, CDCl_3_): δ 160.8 (C=O), 136.2 (Ar), 128.6 (Ar), 126.1 (Ar), 125.3 (Ar), 122.8 (Ar), 119.5 (Ar), 116.4 (Ar), 108.4 (Ar), 74.9 (N(1)OCH_2_), 65.8 (O*C*H_2_CH_3_), 61.9 (C(3)*C*H_2_O), 52.3 (CO_2_*C*H_3_), 15.5 (N(1)OCH_2_*C*H_3_), 13.7 (OCH_2_*C*H_3_); MS *m*/*z* 311 [M]^+^; HRMS (+ESI) calcd for C_15_H_18_ClNNaO_4_ [M + Na]^+^ 334.0822, found 334.0820.

*Methyl 4-Chloro-1-n-propyloxy-3-[(n-propyloxy)methyl]-1H-indole-2-carboxylate (* **1xc** *)*

Use of SnCl_2_·2H_2_O (140 mg, 0.62 mmol, 3.3 eq), *n*-propanol (28 μL, 0.37 mmol, 2.0 eq) and **2x** (50 mg, 0.185 mmol, 1.0 eq) for 1 h at 40 ^°C^, then use of DBU (272 μL, 1.85 mmol, 10.0 eq) and 1-bromopropane (34 μL, 0.37 mmol, 2.0 eq) for 1 h at 25 °C in general procedure afforded the title compound **1xc** (18.9 mg, 30%) as a white oil. Bp 170 °C (decomp.); *R*_f_ 0.54 (1:4 EtOAc/hexanes); HPLC t*_R_* 30.8 min; UV vis (CH_3_CN-H_2_O) λ_max_ 230, 298nm; ^1^H NMR (300 MHz, CDCl_3_): δ 7.35 (d, *J* = 7.9 Hz, 1H, Ar), 7.23 (t, *J* = 8.0 Hz, 1H, Ar), 7.15 (d, *J* = 7.1 Hz, 1H, Ar), 5.09 (s, 2H, C(3)CH_2_O), 4.29 (t, *J* = 6.4 Hz, 2H, N(1)OCH_2_), 3.98 (s, 3H, CO_2_CH_3_), 3.54 (t, *J* = 6.5 Hz, 2H, OCH_2_), 1.86 (sextet, *J* = 6.9 Hz, 2H, N(1)OCH_2_C*H*_2_), 1.64 (sextet, *J* = 7.0 Hz, 2H, OCH_2_C*H*_2_), 1.11 (t, *J* = 7.2 Hz, 3H, N(1)OCH_2_CH_2_C*H*_3_), 0.93 (t, *J* = 7.3 Hz, 3H, OCH_2_CH_2_C*H*_3_); ^13^C NMR (75 MHz, CDCl_3_): δ 160.9 (C=O), 136.1 (Ar), 128.6 (Ar), 126.0 (Ar), 125.3(Ar), 122.8 (Ar), 119.6 (Ar), 116.4 (Ar), 108.3 (Ar), 80.6 (N(1)OCH_2_), 72.4 (OCH_2_), 62.1 (C(3)*C*H_2_O), 52.2 (CO_2_*C*H_3_), 23.1 (N(1)OCH_2_*C*H_2_), 21.8 (OCH_2_*C*H_2_), 10.9 (N(1)O(CH_2_)_2_*C*H_3_), 10.6 (O(CH_2_)_2_*C*H_3_); MS *m*/*z* 339 [M]^+^; HRMS (+ESI) calcd for C_17_H_22_ClNO_4_ [M + Na]^+^ 362.1135, found 362.1134.

*Methyl 4-Chloro-1-n-butyloxy-3-[(n-butyloxy)methyl]-1H-indole-2-carboxylate (* **1xd** *)*

Use of SnCl_2_·2H_2_O (82.8 mg, 0.37 mmol, 3.3 eq), *n*-butanol (21 μL, 0.22 mmol, 2.0 eq) and **2x** (30 mg, 0.11 mmol, 1.0 eq) for 1 h at 40 °C, then use of DBU (165 μL, 1.10 mmol, 10.0 eq) and 1-bromobutane (24 μL, 0.22 mmol, 2.0 eq) for 1 h at 25 °C in general procedure afforded the title compound **1xd** (12.9 mg, 32%) as a white oil. Bp 198 °C (decomp.); *R*_f_ 0.54 (1:4 EtOAc/hexanes); HPLC t*_R_* 34.3 min; UV vis (CH_3_CN-H_2_O) λ_max_ 235, 298 nm; ^1^H NMR (300 MHz, CDCl_3_): δ 7.34 (d, *J* = 8.2 Hz, 1H, Ar), 7.23 (t, *J* = 8.2 Hz, 1H, Ar), 7.15 (d, *J* = 7.4 Hz, 1H, Ar), 5.08 (s, 2H, C(3)CH_2_O), 4.32 (t, *J* = 6.5 Hz, 2H, N(1)OCH_2_), 3.98 (s, 3H, CO_2_CH_3_), 3.58 (t, *J* = 6.5 Hz, 2H, OCH_2_), 1.82 (quintet, *J* = 7.0 Hz, 2H, N(1)OCH_2_C*H*_2_), 1.65–1.52 (m, 4H, OCH_2_C*H*_2_, N(1)OCH_2_CH_2_C*H*_2_), 1.39 (sextet, *J* = 7.5 Hz, 2H, O(CH_2_)_2_C*H*_2_), 1.00 (t, *J* = 7.4 Hz, 3H, N(1)O(CH_2_)_3_C*H*_3_), 0.90 (t, *J* = 7.4 Hz, 3H, O(CH_2_)_3_C*H*_3_); ^13^C NMR (75 MHz, CDCl_3_): δ 160.9 (C=O), 136.1 (Ar), 128.6 (Ar), 126.0 (Ar), 125.3 (Ar), 122.8 (Ar), 119.6 (Ar), 116.4 (Ar), 108.3 (Ar), 79.0 (N(1)OCH_2_), 70.4 (OCH_2_), 62.1 (C(3)*C*H_2_O), 52.3 (CO_2_*C*H_3_), 32.1 (N(1)OCH_2_*C*H_2_), 30.5 (OCH_2_*C*H_2_) 19.6 (N(1)O(CH_2_)_2_*C*H_2_), 19.4 (O(CH_2_)_2_*C*H_2_) 14.2 (N(1)O(CH_2_)_3_*C*H_3_), 14.1 (O(CH_2_)_3_*C*H_3_); MS *m*/*z* 367 [M]^+^; HRMS (+ESI) calcd for C_19_H_26_ClNO_4_ [M + Na]^+^ 390.1448, found 390.1447.

*Methyl 4-Chloro-1-n-pentyloxy-3-[(n-pentyloxy)methyl]-1H-indole-2-carboxylate (* **1xe** *)*

Use of SnCl_2_·2H_2_O (82.8 mg, 0.37 mmol, 3.3 eq), *n*-pentanol (24 μL, 0.22 mmol, 2.0 eq) and **2x** (30 mg, 0.11 mmol, 1.0 eq) for 2 h at 40 °C, then use of DBU (165 μL, 1.10 mmol, 10.0 eq) and 1-bromopentane (28 μL, 0.22 mmol, 2.0 eq) for 1 h at 25 °C in general procedure afforded the title compound **1xe** (8.7 mg, 20%) as a white oil. Bp 184 °C (decomp.); *R*_f_ 0.58 (1:4 EtOAc/hexanes); HPLC t*_R_* 32.9 min; UV vis (CH_3_CN-H_2_O) λ_max_ 235, 298 nm; ^1^H NMR (300 MHz, CDCl_3_): δ 7.34 (d, *J* = 8.1 Hz, 1H, Ar), 7.23 (t, *J* = 8.1 Hz, 1H, Ar), 7.15 (d, *J* = 7.5 Hz, 1H, Ar), 5.08 (s, 2H, C(3)CH_2_O), 4.32 (t, *J* = 6.6 Hz, 2H, N(1)OCH_2_), 3.98 (s, 3H, CO_2_CH_3_), 3.59 (t, *J* = 6.7 Hz, 2H, OCH_2_), 1.84 (quintet, *J* = 7.4 Hz, 2H, N(1)OCH_2_C*H*_2_), 1.64–1.25 (m, 10H, OCH_2_(C*H*_2_)_3_CH_3,_ N(1)OCH_2_CH_2_(C*H*_2_)_2_CH_3_), 0.95 (t, *J* = 7.2 Hz, 3H, N(1)O(CH_2_)_4_C*H*_3_), 0.87 (t, *J* = 6.9 Hz, 3H, O(CH_2_)_4_C*H*_3_); ^13^C NMR (75 MHz, CDCl_3_): δ 160.8 (C=O), 136.0 (Ar), 128.6 (Ar), 126.0 (Ar), 125.3 (Ar), 122.8 (Ar), 119.6 (Ar), 116.4 (Ar), 108.2 (Ar), 79.3 (N(1)OCH_2_), 70.7 (OCH_2_), 62.1 (C(3)*C*H_2_O), 52.2 (CO_2_*C*H_3_), 29.7, 28.6, 28.3, 28.1, 22.7, 22.6, (N(1)OCH_2_(*C*H_2_)_3_, OCH_2_(*C*H_2_)_3_), 14.2 (N(1)O(CH_2_)_4_*C*H_3_), 14.1 (O(CH_2_)_4_*C*H_3_); MS *m*/*z* 395 [M]^+^; HRMS (+ESI) calcd for C_21_H_30_ClNO_4_ [M + Na]^+^ 418.1761, found 418.1760.

*Methyl 4-Chloro-1-n-hexyloxy-3-[(n-hexyloxy)methyl]-1H-indole-2-carboxylate (* **1xf** *)*

Use of SnCl_2_·2H_2_O (140 mg, 0.62 mmol, 3.3 eq), *n*-hexanol (74 μL, 0.37 mmol, 2.0 eq) and **2x** (50 mg, 0.185 mmol, 1.0 eq) for 2 h at 40 °C, then use of DBU (272 μL, 1.85 mmol, 10.0 eq) and 1-bromohexane (52 μL, 0.37 mmol, 2.0 eq) for 1 h at 25 °C in general procedure afforded the title compound **1xf** (16.5 mg, 21%) as a pale-yellow oil. Bp 152 °C (decomp.); *R*_f_ 0.63 (1:4 EtOAc/hexanes); HPLC t*_R_* 29.7 min; UV vis (CH_3_CN-H_2_O) λ_max_ 236, 298 nm; ^1^H NMR (300 MHz, CDCl_3_): δ 7.33 (d, *J* = 8.2 Hz, 1H, Ar), 7.23 (t, *J* = 8.0 Hz, 1H, Ar), 7.14 (d, *J* = 8.0 Hz, 1H, Ar), 5.08 (s, 2H, C(3)CH_2_O), 4.32 (t, *J* = 6.6 Hz, 2H, N(1)OCH_2_), 3.98 (s, 3H, OCH_3_), 3.57 (t, *J* = 6.6 Hz, 2H, OCH_2_), 1.83 (quintet, *J* = 6.7 Hz, 2H, N(1)OCH_2_C*H*_2_), 1.63–1.50 (m, 4H, OCH_2_C*H*_2_, N(1)O(CH_2_)_2_C*H*_2_), 1.37–1.25 (m, 10H, OCH_2_CH_2_(C*H*_2_)_3_, N(1)OCH_2_CH_2_CH_2_(C*H*_2_)_2_), 0.92–0.84 (m, 6H, N(1)O(CH_2_)_5_C*H*_3_, O(CH_2_)_5_C*H*_3_); ^13^C NMR (75 MHz, CDCl_3_): δ 160.7 (C=O), 135.9 (Ar), 128.5 (Ar), 125.8 (Ar), 125.1(Ar), 122.6 (Ar), 119.4 (Ar), 116.2 (Ar), 108.1 (Ar), 79.1 (N(1)OCH_2_), 70.5 (OCH_2_), 61.9 (C(3)*C*H_2_O), 52.0 (CO_2_*C*H_3_), 31.7, 31.6, 29.8, 28.2, 25.9, 25.6, 22.6, 22.5 (N(1)OCH_2_(*C*H_2_)_4_, OCH_2_(*C*H_2_)_4_), 14.1 (N(1)O(CH_2_)_5_*C*H_3_), 14.0 (O(CH_2_)_5_*C*H_3_); MS *m*/*z* 423 [M]^+^; HRMS (+ESI) calcd for C_23_H_34_ClNO_4_ [M + Na]^+^ 446.2074, found 446.2071.

*Methyl 4-Chloro-1-n-octyloxy-3-[(n-octyloxy)methyl]-1H-indole-2-carboxylate (* **1xg** *)*

Use of SnCl_2_·2H_2_O (166 mg, 0.74 mmol, 3.3 eq), *n*-octanol (70 μL, 0.45 mmol, 2.0 eq) and **2x** (60 mg, 0.22 mmol, 1.0 eq) for 2 h at 40 °C, then use of DBU (350 μL, 2.20 mmol, 10.0 eq) and 1-bromooctane (80 μL, 0.45 mmol, 2.0 eq) for 1 h at 25 °C in general procedure afforded the title compound **1xg** (23.2 mg, 22%) as a white solid. Mp 19–20 °C; *R*_f_ 0.70 (1:4 EtOAc/hexanes); HPLC t*_R_* 35.5 min; UV vis (CH_3_CN-H_2_O) λ_max_ 237, 298 nm; ^1^H NMR (300 MHz, CDCl_3_): δ 7.33 (d, *J* = 8.2 Hz, 1H, Ar), 7.23 (t, *J* = 8.1 Hz, 1H, Ar), 7.14 (d, *J* = 7.4 Hz, 1H, Ar), 5.08 (s, 2H, C(3)CH_2_O), 4.32 (t, *J* = 6.6 Hz, N(1)OCH_2_), 3.98 (s, 3H, CO_2_CH_3_), 3.56 (t, *J* = 6.7 Hz, 2H, OCH_2_), 1.83 (quintet, *J* = 7.0 Hz, 2H, N(1)OCH_2_C*H*_2_), 1.66–1.25 (m, 22H, OCH_2_(C*H*_2_)_6_CH_3_), N(1)OCH_2_CH_2_(C*H*_2_)_5_CH_3_), 0.89–0.85 (m, 6H, N(1)O(CH_2_)_7_C*H*_3_, O(CH_2_)_7_C*H*_3_); ^13^C NMR (75 MHz, CDCl_3_): δ 160.8 (C=O), 136.1 (Ar), 128.7 (Ar), 126.0 (Ar), 125.3(Ar), 122.8 (Ar), 120.0 (Ar), 116.4 (Ar), 108.3(Ar), 79.3 (N(1)OCH_2_), 70.7 (OCH_2_), 62.1 (C(3)*C*H_2_O), 52.2 (CO_2_*C*H_3_), 32.0, 31.9, 30.0, 29.9, 29.6, 29.5, 29.4, 28.5, 26.4, 26.2, 22.8, 22.6 5 (N(1)CH_2_(*C*H_2_)_6,_ OCH_2_(*C*H_2_)_6_), 14.3 (N(1)O(CH_2_)_7_*C*H_3_), 13.5 (O(CH_2_)_7_*C*H_3_); MS *m*/*z* 479 [M]^+^; HRMS (+ESI) calcd for C_27_H_42_ClNNaO_4_ [M + Na]^+^ 502.2700, found 502.2926.

*Methyl 4-Chloro-1-benzyloxy-3-[(benzyloxy)methyl]-1H-indole-2-carboxylate (* **1xh** *)*

Use of SnCl_2_·2H_2_O (38.4 mg, 0.17 mmol, 3.3 eq), benzyl alcohol (12 μL, 0.11 mmol, 2.0 eq) and **2x** (14.5 mg, 0.05 mmol, 1.0 eq) for 1 h at 40 °C, then use of DBU (165 μL, 0.55 mmol, 10.0 eq) and benzyl bromide (14 μL, 0.11 mmol, 2.0 eq) for 1 h at 25 °C in general procedure afforded the title compound **1xh** (7.4 mg, 32%) as a white solid. Mp 74 °C; *R*_f_ 0.54 (1:2 EtOAc/hexanes); HPLC t*_R_* 31.8 min; UV vis (CH_3_CN-H_2_O) λ_max_ 212, 298 nm; ^1^H NMR (300 MHz, CDCl_3_): δ 7.50–7.10 (m, 13H, Ar), 5.32 (s, 2H, C(3)CH_2_O), 5.17 (s, 2H, N(1)OCH_2_), 4.66 (s, 2H, C(3)CH_2_OC*H*_2_), 3.87 (s, 3H, CO_2_CH_3_); ^13^C NMR (75 MHz, CDCl_3_): δ 160.8 (C=O), 138.8 (Ar), 136.4 (Ar), 134.3 (Ar), 130.0 (Ar), 129.5 (Ar), 128.9 (Ar), 128.6 (Ar), 128.5 (Ar), 128.2 (Ar), 127.7 (Ar), 126.2 (Ar), 125.9 (Ar), 123.0 (Ar), 119.7 (Ar), 116.2 (Ar), 108.6 (Ar), 81.0 (N(1)OCH_2_), 72.6 (OCH_2_Ph), 61.8 (C(3)*C*H_2_O), 52.2 (CO_2_*C*H_3_); MS *m*/*z* 435 [M]^+^; HRMS (+ESI) calcd for C_25_H_22_ClNNaO_4_ [M + Na]^+^ 458.1135, found 458.1133.

*Methyl 4-Chloro-1-phenylethyloxy-3-[(phenylethyloxy)methyl]-1H-indole-2-carboxylate (* **1xi** *)*

Use of SnCl_2_·2H_2_O (140 mg, 0.62 mmol, 3.3 eq), 2-phenylethyl alcohol (46 μL, 0.37 mmol, 2.0 eq) and **2x** (50 mg, 0.185 mmol, 1.0 eq) for 1 h at 40 °C, then use of DBU (272 μL, 1.85 mmol, 10.0 eq) and 2-(bromoethyl)benzene (51 μL, 0.37 mmol, 2.0 eq) for 1 h at 25 °C in general procedure afforded the title compound **1xi** (15.2 mg, 18%) as a pale yellow solid. Mp 50 °C; *R*_f_ 0.60 (1:2 EtOAc/hexanes); HPLC t*_R_* 34.5 min; UV vis (CH_3_CN-H_2_O) λ_max_ 212, 236, 297 nm; ^1^H NMR (300 MHz, CDCl_3_): δ 7.38–6.90 (m, 13H, Ar), 5.14 (s, 2H, C(3)CH_2_O), 4.55 (t, *J* = 6.7 Hz, 2H, N(1)OCH_2_), 3.86 (s, 3H, CO_2_CH_3_), 3.79 (t, *J* = 7.4 Hz, 2H, C(3)CH_2_OC*H*_2_), 3.14 (t, *J* = 6.7 Hz, 2H, N(1)OCH_2_C*H*_2_), 2.94 (t, *J* = 7.5 Hz, 2H, C(3)CH_2_OCH_2_C*H*_2_); ^13^C NMR (75 MHz, CDCl_3_): δ 160.8 (C=O), 139.2 (Ar), 137.8 (Ar), 136.2 (Ar), 129.3 (Ar), 129.1 (Ar), 128.8 (Ar), 128.5 (Ar), 128.4 (Ar), 126.9 (Ar), 126.2 (Ar), 125.9 (Ar), 125.4 (Ar), 122.9 (Ar), 119.6 (Ar), 116.4 (Ar), 108.3 (Ar), 79.6 (N(1)OCH_2_), 71.4 (OCH_2_), 62.2 (C(3)*C*H_2_O), 52.2 (CO_2_*C*H_3_), 36.5 (N(1)OCH_2_*C*H_2_), 35.0 (OCH_2_*C*H_2_); MS *m*/*z* 463 [M]^+^; HRMS (+ESI) calcd for C_27_H_26_ClNNaO_4_ [M + Na]^+^ 486.1448, found 486.1445.

*Methyl 4-Chloro-1-isopropyloxy-3-[(isopropyloxy)methyl]-1H-indole-2-carboxylate (* **1xj** *)*

Use of SnCl_2_·2H_2_O (166 mg, 0.74 mmol, 3.3 eq), isopropanol (35 μL, 0.45 mmol, 2.0 eq) and **2x** (60 mg, 0.22 mmol, 1.0 eq) for 2 h at 40 °C, then use of DBU (350 μL, 2.20 mmol, 10.0 eq) and 2-bromopropane (43 μL, 0.45 mmol, 2.0 eq) for 2 h at 25 °C in general procedure afforded the title compound **1xj** (9.2 mg, 12%) as a white oil. Bp 164 °C (decomp.); *R*_f_ 0.50 (1:4 EtOAc/hexanes); HPLC t*_R_* 29.1 min; UV vis (CH_3_CN-H_2_O) λ_max_ 233, 298 nm; ^1^H NMR (300 MHz, CDCl_3_): δ 7.35 (d, *J* = 8.2 Hz, 1H, Ar), 7.20 (t, *J* = 7.6 Hz, 1H, Ar), 7.13 (d, *J* = 7.4 Hz, 1H, Ar), 5.09 (s, 2H, C(3)CH_2_O), 4.72 (septet, *J* = 6.2 Hz, 1H, N(1)OC*H*(CH_3_)_2_), 3.80 (septet, 1H, C(3)CH_2_OC*H*(CH_3_)_2_), 1.33 (d, *J* = 6.2 Hz, 6H, N(1)OCH(C*H*_3_)_2_), 1.24 (d, *J* = 6.1 Hz, 6H, C(3)CH_2_OCH(C*H*_3_)_2_); ^13^C NMR (75 MHz, CDCl_3_): δ 161.1 (C=O), 137.5 (Ar), 128.4 (Ar), 126.1 (Ar), 125.8 (Ar), 122.6 (Ar), 119.5 (Ar), 116.8 (Ar), 109.4 (Ar), 82.1 (N(1)OCH), 71.5 (OCH), 60.0 (C(3)*C*H_2_O), 52.2 (CO_2_*C*H_3_), 22.4 (N(1)OCH(*C*H_3_)_2_), 21.3 (OCH(*C*H_3_)_2_); MS *m*/*z* 339 [M]^+^; HRMS (+ESI) calcd for C_17_H_22_ClNNaO_4_ [M + Na]^+^ 362.1135, found 362.1131.

*Methyl 4-Chloro-1-cyclohexyloxy-3-[(cyclohexyloxy)methyl]-1H-indole-2-carboxylate (* **1xk** *)*

Use of SnCl_2_·2H_2_O (82.8 mg, 0.37 mmol, 3.3 eq), cyclohexanol (23 μL, 0.22 mmol, 2.0 eq) and **2x** (30 mg, 0.11 mmol, 1.0 eq) for 2 h at 40 °C, then use of DBU (165 μL, 1.10 mmol, 10.0 eq) and bromocyclohexane (27 μL, 0.22 mmol, 2.0 eq) for 4 h at 50 °C in general procedure afforded the title compound **1xk** (4.7 mg, 11%) as a white solid. Mp 60–64 °C; *R*_f_ 0.57 (1:4 EtOAc/hexanes); HPLC t*_R_* 31.9 min; UV vis (CH_3_CN-H_2_O) λ_max_ 237, 298 nm; ^1^H NMR (300 MHz, CDCl_3_): δ 7.37 (d, *J* = 8.2 Hz, 1H, Ar), 7.20 (t, *J* = 7.6 Hz, 1H, Ar), 7.12 (d, *J* = 7.5 Hz, 1H, Ar), 5.11 (s, 2H, C(3)CH_2_O), 4.35–4.28 (m, 1H, N(1)OCH), 3.97 (s, 3H, CO_2_CH_3_), 3.45–3.38 (m, 1H, C(3)CH_2_OC*H*), 2.38–0.86 (m, 20H, N(1)OCH(C*H*_2_)_5_, C(3)CH_2_OCH(C*H*_2_)_5_); ^13^C NMR (75 MHz, CDCl_3_): δ 161.1 (C=O), 137.3 (Ar) 128.4 (Ar), 126.0 (Ar), 125.7 (Ar), 122.5 (Ar), 119.2 (Ar), 116.7 (Ar), 109.4 (Ar), 87.9 (N(1)OCH), 78.3 (C(3)CH_2_O*C*H), 59.7 (C(3)*C*H_2_O), 52.2 (CO_2_*C*H_3_), 31.7, 29.9. 26.1, 25.6, 24.7, 24.6 (N(1)OCH(*C*H_2_)_5_, OCH(*C*H_2_)_5_); MS *m*/*z* 419 [M]^+^; HRMS (+ESI) calcd for C_23_H_30_ClNNaO_4_ [M + Na]^+^ 442.1760, found 442.1760.

*Methyl 4-Chloro-1-methoxy-3-[(phenylethyloxy)methyl]-1H-indole-2-carboxylate (* **1xl** *)*

Use of SnCl_2_·2H_2_O (140 mg, 0.62 mmol, 3.3 eq), 2-phenylethyl alcohol (46 μL, 0.37 mmol, 2.0 eq) and **2x** (50 mg, 0.185 mmol, 1.0 eq) for 2 h at 40 °C, then use of DBU (272 μL, 1.85 mmol, 10.0 eq) and methyl iodide (23 μL, 0.37 mmol, 2.0 eq) for 2 h at 25 °C in general procedure afforded the title compound **1xl** (15.2 mg, 22%) as a white solid. Mp 68 °C; *R*_f_ 0.30 (1:4 EtOAc/hexanes); HPLC t*_R_* 29.0 min; UV vis (CH_3_CN-H_2_O) λ_max_ 215, 234, 298 nm; ^1^H NMR (300 MHz, CDCl_3_): δ 7.38 (d, *J* = 8.2 Hz, 2H, Ar), 7.29–7.16 (m, 6H, Ar), 5.15 (s, 2H, C(3)CH_2_O,), 4.19 (s, 3H, N(1)OCH_3_), 3.96 (s, 3H, CO_2_CH_3_), 3.81 (t, *J* = 7.4 Hz, 2H, OCH_2_), 2.95 (t, *J* = 7.4 Hz, 2H, OCH_2_C*H*_2_); ^13^C NMR (75 MHz, CDCl_3_): δ 160.8 (C=O), 139.3 (Ar), 135.9 (Ar), 129.1 (Ar), 128.7 (Ar), 128.5 (Ar), 126.3 (Ar), 126.2 (Ar), 125.3 (Ar), 123.0 (Ar), 119.7 (Ar), 116.5 (Ar), 108.2 (Ar), 71.5 (N(1)OCH_3_), 66.4 (OCH_2_), 62.2 (C(3)*C*H_2_O), 52.4 (CO_2_*C*H_3_), 36.5 (OCH_2_*C*H_2_); MS *m*/*z* 373 [M]^+^; HRMS (+ESI) calcd for C_20_H_20_ClNNaO_4_ [M + Na]^+^ 396.0979, found 396.0977.

*Methyl 4-Chloro-1-n-octyloxy -3-[(methoxymethyl]-1H-indole-2-carboxylate (* **1xm** *)*

Use of SnCl_2_·2H_2_O (82.8 mg, 0.37 mmol, 3.3 eq), methanol (9 μL, 0.22 mmol, 2.0 eq) and **2x** (30 mg, 0.11 mmol, 1.0 eq) for 2 h at 40 °C, then use of DBU (165 μL, 1.10 mmol, 10.0 eq) and 1-bromooctane (38 μL, 0.22 mmol, 2.0 eq) for 2 h at 25 °C in general procedure afforded the title compound **1xm** (13.7 mg, 33%) as a yellow oil. Bp 178 °C (decomp.); *R*_f_ 0.67 (1:2 EtOAc/hexanes); HPLC t*_R_* 26.3 min; UV vis (CH_3_CN-H_2_O) λ_max_ 236, 298 nm; ^1^H NMR (300 MHz, CDCl_3_): δ 7.36 (dd, *J* = 8.2, 1.0 Hz, 1H, Ar), 7.25 (t, *J* = 7.8 Hz, 1H, Ar), 7.16 (dd, *J* = 7.5, 1.0 Hz, 1H, Ar), 5.08 (s, 2H, C(3)CH_2_O,), 4.32 (t, *J* = 6.6 Hz, 2H, N(1)OCH_2_), 4.00 (s, 3H, CO_2_CH_3_), 3.46 (s, 3H, OCH_3_), 1.83 (quintet, *J* = 7.1 Hz, 2H, OCH_2_C*H*_2_), 1.54–1.47 (m, 2H, O(CH_2_)_2_C*H*_2_), 1.34–1.25 (m, 8H, O(CH_2_)_3_(C*H*_2_)_4_, 0.90 (t, *J* = 7.0 Hz, 3H, O(CH_2_)_7_C*H*_3_); ^13^C NMR (75 MHz, CDCl_3_): δ 160.8 (C=O), 136.0 (Ar), 128.5 (Ar), 126.1 (Ar), 125.2 (Ar), 122.9 (Ar), 119.5 (Ar), 116.1 (Ar), 108.4 (Ar), 79.4 (N(1)OCH_2_), 63.7 (OCH_3_), 58.1 (C(3)*C*H_2_O), 52.3 (CO_2_*C*H_3_), 32.0, 29.6, 29.4, 28.4, 26.2, 22.8 (N(1)OCH_2_(*C*H_2_)_6_), 14.3 N(1)O(CH_2_)_7_*C*H_3_; MS *m*/*z* 381 [M]^+^; HRMS (+ESI) calcd for C_20_H_28_ClNO_4_ [M]^+^ 381.1707, found 381.1707.

*Methyl 4-Chloro-1-methoxy-3-[(n-octyloxy)methyl]-1H-indole-2-carboxylate (* **1xn** *)*

Use of SnCl_2_·2H_2_O (82.8 mg, 0.37 mmol, 3.3 eq), *n*-octanol (35 μL, 0.22 mmol, 2.0 eq) and **2x** (30 mg, 0.11 mmol, 1.0 eq) for 2 h at 40 °C, then use of DBU (165 μL, 1.10 mmol, 10.0 eq) and methyl iodide (14 μL, 0.22 mmol, 2.0 eq) for 2 h at 25 °C in general procedure afforded the title compound **1xn** (15.0 mg, 36%) as a white solid. Mp 36 °C; *R*_f_ 0.74 (1:2 EtOAc/hexanes); HPLC t*_R_* 27.0 min; UV vis (CH_3_CN-H_2_O) λ_max_ 236, 298 nm; ^1^H NMR (300 MHz, CDCl_3_): δ 7.37 (d, *J* = 8.2 Hz, 1H, Ar), 7.26 (t, *J* = 7.8 Hz, 1H, Ar), 7.16 (d, *J* = 7.1 Hz, 1H, Ar), 5.08 (s, 2H, C(3)CH_2_O,), 4.19 (s, 3H, N(1)OCH_3_), 4.00 (s, 3H, CO_2_CH_3_), 3.57 (t, *J* = 6.7 Hz, 2H, OCH_2_), 1.66–1.59 (m, 2H, OCH_2_C*H*_2_), 1.43–1.25 (m, 10H, O(CH_2_)_2_(C*H*_2_)_5_), 0.87 (t, *J* = 6.6 Hz, 3H, O(CH_2_)_7_C*H*_3_); ^13^C NMR (75 MHz, CDCl_3_): δ 160.9 (C=O), 136.0 (Ar), 128.8 (Ar), 126.3 (Ar), 125.3 (Ar), 122.9 (Ar), 119.7 (Ar), 116.7 (Ar), 108.1 (Ar), 70.8 (N(1)OCH_3_), 66.4 (OCH_2_), 62.1 (C(3)*C*H_2_O), 52.4 (CO_2_*C*H_3_), 32.0, 30.0, 29.6, 29.5, 26.4, 22.9 (OCH_2_(*C*H_2_)_6_), 14.3 (O(CH_2_)_7_*C*H_3_); MS *m*/*z* 381 [M]^+^; HRMS (+ESI) calcd for C_20_H_28_ClNO_4_ [M]^+^ 381.1707, found 381.1707.

*Methyl 4-Bromo-1-methoxy-3-(methoxymethyl)-1H-indole-2-carboxylate (* **1ya** *)*

Use of SnCl_2_·2H_2_O (75 mg, 0.33 mmol, 3.3 eq), methanol (8 μL, 0.20 mmol, 2.0 eq) and **2y** (31.4 mg, 0.10 mmol, 1.0 eq) for 2 h at 40 °C, then use of DBU (150 μL, 1.00 mmol, 10.0 eq) and methyl iodide (13 μL, 0.20 mmol, 2.0 eq) for 2 h at 25 °C in general procedure afforded the title compound **1ya** (17.1 mg, 52%) as a white solid. Mp 50–52 °C; *R*_f_ 0.29 (1:4 EtOAc/hexanes); HPLC t*_R_* 34.3 min; UV vis (CH_3_CN-H_2_O) λ_max_ 215, 298 nm; ^1^H NMR (300 MHz, CDCl_3_): δ 7.49–7.38 (m, 2H, Ar), 7.19 (t, *J* = 8.1 Hz, 1H, Ar), 5.08 (s, 2H, C(3)CH_2_O), 4.18 (s, 3H, N(1)OCH_3_), 4.01 (s, 3H, CO_2_CH_3_), 3.47 (s, 3H, CH_2_OC*H*_3_); ^13^C NMR (75 MHz, CDCl_3_): δ 160.8 (C=O), 135.8 (Ar), 131.2 (Ar), 126.7 (Ar), 125.6 (Ar), 123.8 (Ar), 120.9 (Ar), 116.7 (Ar), 108.8 (Ar), 66.4 (N(1)OCH_3_), 63.0 (CH_2_O*C*H_3_), 58.0 (C(3)*C*H_2_O), 52.4 (CO_2_*C*H_3_); MS *m*/*z* 327 [M]^+^; HRMS (+ESI) calcd for C_13_H_14_BrNNaO_4_ [M + Na]^+^ 350.0004, found 350.0002.

*Methyl 4-Bromo-1-ethoxy-3-(ethoxymethyl)-1H-indole-2-carboxylate (* **1yb** *)*

Use of SnCl_2_·2H_2_O (75 mg, 0.33 mmol, 3.3 eq), ethanol (12 μL, 0.20 mmol, 2.0 eq) and **2y** (31.4 mg, 0.10 mmol, 1.0 eq) for 2 h at 40 °C, then use of DBU (150 μL, 1.00 mmol, 10.0 eq) and bromoethane (16 μL, 0.20 mmol, 2.0 eq) for 2 h at 25 °C in general procedure afforded the title compound **1yb** (9.3 mg, 26%) as a white solid. Mp 42 °C; *R*_f_ 0.51 (1:2 EtOAc/hexanes); HPLC t*_R_* 26.2 min; UV vis (CH_3_CN-H_2_O) λ_max_ 235, 299 nm; ^1^H NMR (300 MHz, CDCl_3_): δ 7.45–7.33 (m, 2H, Ar), 7.16 (t, *J* = 7.9 Hz, 1H, Ar), 5.10 (s, 2H, C(3)CH_2_O), 4.41 (q, *J* = 7.1 Hz, 2H, N(1)OCH_2_), 3.99 (s, 3H, CO_2_CH_3_), 3.66 (q, *J* = 7.0 Hz, 2H, OCH_2_), 1.44 (t, *J* = 7.1 Hz, 3H, N(1)OCH_2_C*H*_3_), 1.26 (t, *J* = 7.0 Hz 3H, OCH_2_C*H*_3_), ^13^C NMR (75 MHz, CDCl_3_): δ 160.9 (C=O), 136.2 (Ar), 126.5 (Ar), 126.3 (Ar), 120.8 (Ar), 125.5(Ar), 116.7 (Ar), 116.4 (Ar), 109.0 (Ar), 75.0 (N(1)OCH_2_), 65.8 (OCH_2_), 61.4 (C(3)*C*H_2_O), 52.3 (CO_2_*C*H_3_), 15.6 (N(1)OCH_2_*C*H_3_), 13.8 (OCH_2_*C*H_3_); MS *m*/*z* 355 [M]^+^; HRMS (+ESI) calcd for C_15_H_19_BrNNaO_4_ [M + Na]^+^ 378.0317, found 378.0315.

*Methyl 4-Bromo-1-n-propyloxy-3-[(n-propyloxy)methyl]-1H-indole-2-carboxylate (* **1yc** *)*

Use of SnCl_2_·2H_2_O (75 mg, 0.33 mmol, 3.3 eq), *n*-propanol (15 μL, 0.20 mmol, 2.0 eq) and **2y** (31.4 mg, 0.10 mmol, 1.0 eq) for 1 h at 40 °C, then use of DBU (150 μL, 1.00 mmol, 10.0 eq) and 1-bromopropane (18 μL, 0.20 mmol, 2.0 eq) for 2 h at 25 °C in general procedure afforded the title compound **1yc** (10.9 mg, 28%) as a white oil. Bp 224 °C (decomp.); *R*_f_ 0.62 (1:2 EtOAc/hexanes); HPLC t*_R_* 31.3 min; UV vis (CH_3_CN-H_2_O) λ_max_ 236, 299 nm; ^1^H NMR (300 MHz, CDCl_3_): δ 7.40 (d, *J* = 8.3 Hz, 1H, Ar), 7.39 (d, *J* = 8.3 Hz, 1H, Ar), 7.15 (t, *J* = 7.7 Hz, 1H, Ar), 5.10 (s, 2H, C(3)CH_2_O), 4.29 (t, *J* = 6.6 Hz, 2H, N(1)OCH_2_), 3.98 (s, 3H, CO_2_CH_3_), 3.55 (t, *J* = 6.6 Hz, 2H, OCH_2_), 1.86 (sextet, *J* = 7.2 Hz, 2H, N(1)OCH_2_C*H*_2_), 1.66 (sextet, *J* = 7.2 Hz, 2H, OCH_2_C*H*_2_), 1.11 (t, *J* = 7.4 Hz, 3H, N(1)OCH_2_CH_2_C*H*_3_), 0.94 (t, *J* = 7.4 Hz, 3H, OCH_2_CH_2_C*H*_3_); ^13^C NMR (75 MHz, CDCl_3_): δ 160.9 (C=O), 136.0 (Ar), 126.5 (Ar), 126.3 (Ar), 125.7(Ar), 120.9 (Ar), 116.8 (Ar), 116.4 (Ar), 108.9 (Ar), 80.7 (N(1)OCH_2_), 72.3 (OCH_2_), 61.5 (C(3)*C*H_2_O), 52.3 (CO_2_*C*H_3_), 23.2 (N(1)OCH_2_*C*H_2_), 21.8 (OCH_2_*C*H_2_), 11.0 (N(1)O(CH_2_)_2_*C*H_3_), 10.7 (O(CH_2_)_2_*C*H_3_); MS *m*/*z* 383 [M]^+^; HRMS (+ESI) calcd for C_17_H_22_BrNNaO_4_ [M + Na]^+^ 406.0630, found 408.0608.

*Methyl 4-Bromo-1-n-octyloxy-3-[(n-octyloxy)methyl]-1H-indole-2-carboxylate (* **1yg** *)*

Use of SnCl_2_·2H_2_O (75 mg, 0.33 mmol, 3.3 eq), *n*-octanol (9 μL, 0.20 mmol, 2.0 eq) and **2y** (31.4 mg, 0.10 mmol, 1.0 eq) for 1 h at 40 °C, then use of DBU (150 μL, 1.00 mmol, 10.0 eq) and 1-bromooctane (35 μL, 0.20 mmol, 2.0 eq) for 2 h at 25 °C in general procedure afforded the title compound **1yg** (11.0 mg, 21%) as a yellow oil. Bp 194 °C (decomp.); *R*_f_ 0.40 (1:10 EtOAc/hexanes); HPLC t*_R_* 36.1 min; UV vis (CH_3_CN-H_2_O) λ_max_ 236, 299 nm; ^1^H NMR (300 MHz, CDCl_3_): δ 7.37 (t, *J* = 7.9 Hz, 2H, Ar), 7.16 (t, *J* = 7.9 Hz, 1H, Ar), 5.09 (s, 2H, C(3)CH_2_O), 4.32 (t, *J* = 6.6 Hz, 2H, N(1)OCH_2_), 3.98 (s, 3H, CO_2_CH_3_), 3.57 (t, *J* = 6.6 Hz, 2H, OCH_2_), 1.83 (quintet, *J* = 7.5 Hz, 2H, N(1)OCH_2_C*H*_2_), 1.66–1.15 (m, 22H, N(1)OCH_2_CH_2_(C*H*_2_)_5_, OCH_2_(C*H*_2_)_6_), 1.00–0.77 (m, 6H, N(1)O(CH_2_)_7_C*H*_3_, O(CH_2_)_7_C*H*_3_); ^13^C NMR (75 MHz, CDCl_3_): δ 160.9 (C=O), 136.0 (Ar), 126.4 (Ar), 126.3 (Ar), 125.6 (Ar), 120.9 (Ar), 116.8 (Ar), 116.4 (Ar), 108.9(Ar), 79.4 (N(1)O*C*H_2_), 70.6 (O*C*H_2_), 61.5 (C(3)*C*H_2_O), 53.3 (CO_2_*C*H_3_), 53.3, 52.3, 32.0, 30.1, 30.0, 29.7, 29.5, 29.4, 28.5, 26.5, 26.2, 22.9 (N(1)OCH_2_(*C*H_2_)_6_,OCH_2_(*C*H_2_)_6_), 14.3 (N(1)O(CH_2_)_7_*C*H_3_), 13.5 (O(CH_2_)_7_*C*H_3_); MS *m*/*z* 523 [M]^+^; HRMS (+ESI) calcd for C_27_H_42_BrNNaO_4_ [M + Na]^+^ 546.2195, found 546.2190.

*Methyl 4-Bromo-1-benzyloxy-3-[(benzyloxy)methyl]-1H-indole-2-carboxylate (* **1yh** *)*

Use of SnCl_2_·2H_2_O (38 mg, 0.17 mmol, 3.3 eq), benzyl alcohol (11 μL, 0.10 mmol, 2.0 eq) and **2y** (15.7 mg, 0.05 mmol, 1.0 eq) for 2 h at 40 °C, then use of DBU (75 μL, 0.50 mmol, 10.0 eq) and benzyl bromide (12 μL, 0.10 mmol, 2.0 eq) for 2 h at 25 °C in general procedure afforded the title compound **1yh** (9.8 mg, 41%) as a pale-yellow solid. Mp 84 °C; *R*_f_ 0.29 (1:2 EtOAc/hexanes); HPLC t*_R_* 33.3 min; UV vis (CH_3_CN-H_2_O) λ_max_ 228, 299 nm; ^1^H NMR (300 MHz, CDCl_3_): δ 7.55–7.22 (m, 12H, Ar), 7.13 (t, *J* = 7.9 Hz, 1H, Ar), 5.32 (s, 2H, C(3)C*H*_2_O), 5.19 (s, 2H, N(1)OCH_2_), 4.68 (s, 2H, OCH_2_), 3.88 (s, 3H, CO_2_CH_3_); ^13^C NMR (75 MHz, CDCl_3_): δ 160.8 (C=O), 138.8 (Ar), 136.3 (Ar), 134.3 (Ar), 130.0 (Ar), 129.5 (Ar), 129.0 (Ar), 128.5 (Ar), 128.3 (Ar), 127.7 (Ar), 126.7 (Ar), 126.4 (Ar), 126.2 (Ar), 121.0 (Ar), 116.5 (Ar), 116.3 (Ar) 109.2 (Ar), 81.0 (N(1)OCH_2_), 72.6 (OCH_2_Ph), 61.2(C(3)*C*H_2_O), 52.3 (CO_2_*C*H_3_); MS *m*/*z* 479 [M]^+^; HRMS (+ESI) calcd for C_25_H_22_BrNNaO_4_ [M + Na]^+^ 502.0630, found 502.0626.

*Methyl 4-Bromo-1-isopropyloxy-3-[(isopropyloxy)methyl]-1H-indole-2-carboxylate (* **1yj** *)*

Use of SnCl_2_·2H_2_O (75 mg, 0.33 mmol, 3.3 eq), isopropanol (15 μL, 0.20 mmol, 2.0 eq) and **2y** (31.4 mg, 0.10 mmol, 1.0 eq) for 2 h at 40 °C, then use of DBU (150 μL, 1.00 mmol, 10.0 eq) and 2-bromopropane (19 μL, 0.20 mmol, 2.0 eq) for 2 h at 25 °C in general procedure afforded the title compound **1yj** (6.2 mg, 16%) as a white oil. Bp 178 °C (decomp.); *R*_f_ 0.69 (1:2 EtOAc/hexanes); HPLC t*_R_* 30.5 min; UV vis (CH_3_CN-H_2_O) λ_max_ 235, 299 nm; ^1^H NMR (300 MHz, CDCl_3_): δ 7.40 (d, *J* = 8.3 Hz, 1H, Ar), 7.35 (d, *J* = 7.5 Hz, 1H, Ar), 7.13 (t, *J* = 7.9 Hz, 1H, Ar), 5.11 (s, 2H, C(3)CH_2_O), 4.72 (septet, *J* = 6.2 Hz, 1H, N(1)OC*H*(CH_3_)_2_), 3.97 (s, 3H, CO_2_CH_3_), 3.82 (septet, *J* = 6.0 Hz, 1H, C(3)CH_2_OC*H*(CH_3_)_2_), 1.34 (d, *J* = 6.2 Hz, 6H, N(1)OCH(C*H*_3_)_2_), 1.26 (d, *J* = 6.1 Hz, 6H, OCH(C*H*_3_)_2_); ^13^C NMR (75 MHz, CDCl_3_): δ 161.1 (C=O), 137.3 (Ar) 126.3 (Ar), 126.1 (Ar), 120.7 (Ar), 117.1 (Ar), 116.1 (Ar), 109.9 (Ar), (one Ar peak was not detected and believed to overlap with the observed peak), 82.2 (N(1)O*C*H(CH_3_)_2_), 71.5 (O*C*H(CH_3_)_2_), 59.4 (C(3)O*C*H_2_), 52.2 (CO_2_*C*H_3_), 22.4 (N(1)OCH(*C*H_3_)_2_), 21.3 (OCH(*C*H_3_)_2_); MS *m*/*z* 383 [M]^+^; HRMS (+ESI) calcd for C_17_H_22_BrNNaO_4_ [M + Na]^+^ 406.0630, found 406.0632.

*Methyl 4-Bromo-1-methoxy-3-[(phenylethyloxy)methyl]-1H-indole-2-carboxylate (* **1yl** *)*

Use of SnCl_2_·2H_2_O (37 mg, 0.17 mmol, 3.3 eq), 2-phenylethyl alcohol (12 μL, 0.10 mmol, 2.0 eq) and **2y** (15.7 mg, 0.05 mmol, 1.0 eq) for 2 h at 40 °C, then use of DBU (75 μL, 0.50 mmol, 10.0 eq) and methyl iodide (4 μL, 0.10 mmol, 2.0 eq) for 2 h at 25 °C in general procedure afforded the title compound **1yl** (5.2 mg, 25%) as a white solid. Mp 59 °C; *R*_f_ 0.57 (1:2 EtOAc/hexanes); HPLC t*_R_* 29.7 min; UV vis (CH_3_CN-H_2_O) λ_max_ 235, 299 nm; ^1^H NMR (300 MHz, CDCl_3_): δ 7.43 (d, *J* = 8.3 Hz, 1H, Ar), 7.38 (d, *J* = 7.4 Hz, 1H, Ar), 7.29–7.16 (m, 6H, Ar), 5.15 (s, 2H, C(3)C*H*_2_O), 4.19 (s, 3H, N(1)OCH_3_), 3.96 (s, 3H, CO_2_CH_3_), 3.82 (t, *J* = 7.4 Hz, 2H, OCH_2_), 2.96 (t, *J* = 7.4 Hz, 2H, OCH_2_C*H*_2_); ^13^C NMR (75 MHz, CDCl_3_): δ 160.8 (C=O), 139.3 (Ar), 135.9 (Ar), 129.1 (Ar), 128.5 (Ar), 126.7 (Ar), 126.5 (Ar), 126.3 (Ar), 125.6 (Ar), 120.9 (Ar), 116.8 (Ar), 116.4 (Ar), 108.8 (Ar), 71.5 (N(1)OCH_3_), 66.4 (OCH_2_), 61.6 (C(3)*C*H_2_O), 52.4 (CO_2_*C*H_3_), 36.5 (OCH_2_*C*H_2_); MS *m*/*z* 419 [M]^+^; HRMS (+ESI) calcd for C_20_H_20_BrNNaO_4_ [M + Na]^+^ 440.0473, found 440.0471.

*Methyl 4-Bromo-1-pentyloxy-3-[(phenylethyloxy)methyl]-1H-indole-2-carboxylate (* **1ym** *)*

Use of SnCl_2_·2H_2_O (37 mg, 0.17 mmol, 3.3 eq), 2-phenylethyl alcohol (12 *μ*L, 0.10 mmol, 2.0 eq), and **2y** (15.7 mg, 0.05 mmol, 1.0 eq) for 2 h at 40 °C, then use of DBU (75 *μ*L, 0.50 mmol, 10.0 eq) and 1-bromopentane (13 *μ*L, 0.10 mmol, 2.0 eq) for 2 h at 25 °C in general procedure afforded the title compound **1ym** (5.0 mg, 21%) as a white oil. Bp 224 °C (decomp.); *R*_f_ 0.70 (1:2 EtOAc/hexanes); HPLC t*_R_* 36.7 min; UV vis (CH_3_CN-H_2_O) λ_max_ 236, 299 nm; ^1^H NMR (300 MHz, CD_3_CN): δ 7.38 (t, *J* = 8.1 Hz, 2H, Ar), 7.30–7.10 (m, 6H, Ar), 5.16 (s, 2H, C(3)CH_2_O), 4.32 (t, *J* = 6.6 Hz, 2H, N(1)OCH_2_), 3.95 (s, 3H, CO_2_CH_3_), 3.81 (t, *J* = 7.5 Hz, 2H, OC*H*_2_CH_2_Ph), 2.96 (t, *J* = 7.4 Hz, 2H, OCH_2_C*H*_2_Ph), 1.95–1.73 (m, 2H, N(1)OCH_2_C*H*_2_), 1.58–1.30 (m, 4H, N(1)OCH_2_CH_2_(C*H*_2_)_2_), 0.96 (t, *J* = 7.1 Hz, 3H, N(1)O(CH_2_)_4_C*H*_3_); ^13^C NMR (75 MHz, CD_3_CN): δ 160.7 (C=O), 139.3 (Ar), 136.0 (Ar), 129.1 (Ar), 128.5 (Ar), 126.5 (Ar), 126.4 (Ar), 126.3 (Ar), 125.6 (Ar), 120.8 (Ar), 116.5 (Ar), 116.4 (Ar), 108.9 (Ar), 79.4 (N(1)OCH_2_), 71.4 (O*C*H_2_CH_2_Ph), 61.6 (C(3)*C*H_2_O), 52.3 (CO_2_*C*H_3_), 36.6 (OCH_2_*C*H_2_Ph), 28.3 (N(1)OCH_2_*C*H_2_), 28.2 (N(1)O(CH_2_)_2_*C*H_2_), 22.8 (N(1)O(CH_2_)_3_*C*H_2_), 14.2 (N(1)O(CH_2_)_4_*C*H_3_); MS *m*/*z* 473 [M]^+^; HRMS (+ESI) calcd for C_24_H_28_BrNNaO_4_ [M+Na]^+^ 496.1099, found 495.1097.

*Methyl 2-(4′-Chloro-1′-hydroxy-1′H-indol-3′-yl)-2-oxoacetate (* **10x** *) [19]*

Brown solid. Mp 84–86 °C; *R*_f_ 0.15 (2:1 EtOAc/hexanes); ^1^H NMR (300 MHz, CD_3_CN): δ 9.45 (br s, 1H, OH), 8.27 (s, 1H), 7.51 (dd, *J* = 5.0, 1.0 Hz, 1H), 7.40 (t, *J* = 4.7 Hz, 1H), 7.33 (dd, *J* = 4.2, 1.0 Hz, 1H), 3.90 (s, 3H); ^13^C NMR (75 MHz, CDCl_3_): δ 179.5, 164.3, 137.5, 127.2, 126.5, 126.4, 126.1, 117.4, 110.0, 109.5, 53.8; MS *m*/*z* 276 [M + Na]^+^; HRMS (+ESI) Calcd for C_11_H_8_ClNNaO_4_ [M + Na]^+^ 276.0040, found 276.0034.

*Methyl 2-(4′-Chloro-1′-methoxy-1′H-indol-3′-yl)-2-oxoacetate (* **11xl** *)*

Use of SnCl_2_·2H_2_O (140 mg, 0.62 mmol, 3.3 eq), 2-phenylethyl alcohol (46 μL, 0.37 mmol, 2.0 eq), and **2x** (50 mg, 0.185 mmol, 1.0 eq) for 2 h at 40 °C, then use of DBU (272 *μ*L, 1.85 mmol, 10.0 eq) and methyl iodide (23 μL, 0.37 mmol, 2.0 eq) for 2 h at 25 °C in general procedure afforded the compound **11xl** (15.8 mg, 32%) as a brown solid. Mp 64 °C; *R*_f_ 0.50 (2:1 EtOAc/hexanes); HPLC t*_R_* 16.6 min; UV vis (CH_3_CN-H_2_O) λ_max_ 218, 262, 321 nm; ^1^H NMR (300 MHz, CDCl_3_): δ 8.40 (s, 1H, C(2)H), 7.43–7.15 (m, 3H, Ar), 4.19 (s, 3H, N(1)OCH_3_), 3.95 (s, 3H, OCH_3_); ^13^C NMR (75 MHz, CDCl_3_): δ 177.7 (C(3)*C*=O), 164.1 (C=O), 134.3 (Ar), 133.5 (Ar), 128.1 (Ar), 125.4 (Ar), 125.3 (Ar), 120.7 (Ar), 109.6 (Ar), 107.8 (Ar), 67.6 (N(1)O*C*H_3_), 53.2 (CO_2_*C*H_3_); MS *m*/*z* 267 [M]^+^; HRMS (+ESI) calcd for C_12_H_10_ClNNaO_4_ [M + Na]^+^ 290.0196, found 290.0193.

*Methyl 2-(4′-Chloro-1′-octyloxy-1′H-indol-3′-yl)-2-oxoacetate (* **11xm** *)*

Use of SnCl_2_·2H_2_O (82.8 mg, 0.37 mmol, 3.3 eq), methanol (9 μL, 0.22 mmol, 2.0 eq), and **2x** (30 mg, 0.11 mmol, 1.0 eq) for 2 h at 40 °C, then use of DBU (165 μL, 1.10 mmol, 10.0 eq) and 1-bromooctane (38 μL, 0.22 mmol, 2.0 eq) for 2 h at 25 °C in general procedure afforded the compound **11xm** (1.1 mg, 3%) as a brown solid. Mp 40 °C; *R*_f_ 0.57 (1:2 EtOAc/hexanes); HPLC t*_R_* 33.4 min; UV vis (CH_3_CN-H_2_O) λ_max_ 219, 262, 320 nm; ^1^H NMR (300 MHz, CDCl_3_): δ 8.37 (s, 1H, C(2)H), 7.40–7.15 (m, 3H, Ar), 4.31 (t, *J* = 6.7 Hz, 2H, N(1)OCH_2_), 3.95 (s, 3H, OCH_3_), 1.82 (quintet, *J* = 7.1 Hz, 2H, N(1)OCH_2_C*H*_2_), 1.57–1.25 (m, 10H, O(CH_2_)_2_(C*H*_2_)_5_, 0.90 (t, *J* = 6.6 Hz, 3H, O(CH_2_)_7_C*H*_3_); ^13^C NMR (75 MHz, CDCl_3_): δ 177.6 (C(3)*C*=O), 164.2 (C=O), 134.9 (Ar), 134.2 (Ar), 128.1 (Ar), 125.4 (Ar), 125.2 (Ar), 109.4 (Ar), 108.0 (Ar), (one Ar peak was not detected and believed to overlap with the observed peak), 80.5 (N(1)OCH_2_), 53.1 (CO_2_*C*H_3_), 32.1, 29.9, 29.5, 28.3, 25.9, 22.8 (N(1)OCH_2_(*C*H_2_)_6_), 14.3 (N(1)O(CH_2_)_7_*C*H_3_); MS *m*/*z* 365 [M]^+^; HRMS (+ESI) calcd for C_19_H_24_ClNO_4_ [M]^+^ 365.1394, found 365.1394.

## 4. Conclusions

We reported the studies on one-pot synthesis of novel multisubstituted 1-alkoxyindoles **1** through four step reactions. With substrates **2** obtained by two-step synthetic sequences, we performed the reactions using SnCl_2_·2H_2_O as a reducing agent and alcohols (R^1^OH) as nucleophiles, through reduction, condensation, and 1,5-addition, affording the intermediates, 1-hydroxyindoles **8**. Subsequent alkylation reactions of **8** using alkyl halides (R^2^Y) in basic condition gave target compounds, 1-alkoxyindoles **1**. The optimized condition was established as follows: 1) conjugate ketoester (1.0 eq), SnCl_2_·2H_2_O (3.3 eq), and alcohols (2.0 eq) in DME for 1–2 h at 40 °C and 2) DBU (10.0 eq) and alkyl halides (2.0 eq) for 1–4 h at 25–50 °C. Considering the yields and reaction efficiency, we chose 2.0 eq of both alcohols and alkyl halides, focusing on the optimization of the final alkylation step. All four step reactions were performed in one-pot, providing 1-alkoxyindoles **1** in modest to good yields (22 examples, 11–52% yields for four steps). Mechanistic investigations on reaction pathways (Path A, B, and C) were presented along with the formation of side products **11**.

## Figures and Tables

**Table 1:**
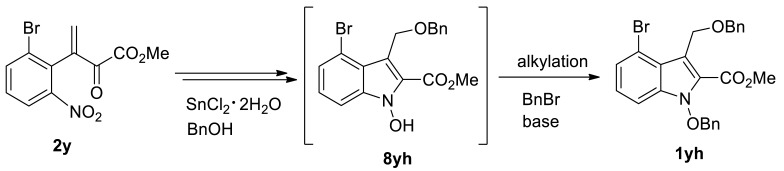
Synthesis of 1-alkoxyindole **1yh** in different base conditions ^a^.

Entry	Base	Yield (%)
1	TEA	23
2	DIEA	17
3	DMAP	13
4	DBU	41
5	K_2_CO_3_^b^	5

^a^ All reactions were run in the 0.05 mmol scale of conjugate ketoester **2y** (1.0 eq, [c] = 0.12 M) and BnOH (2.0 eq) for formation of **8yh** in DME for 2 h at 40 °C; base (10 eq) and BnBr (2.0 eq) for 2 h at 25 °C for formation of **1yh**. ^b^Aqueous K_2_CO_3_ was used with acetone.

**Table 2:**
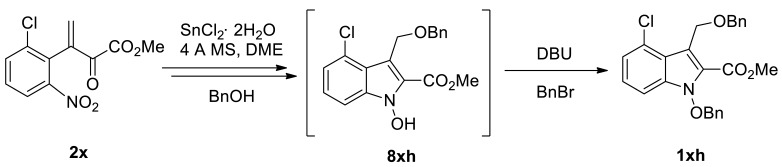
Optimization of the reaction conditions for **1xh** ^a^.

Entry	SnCl_2_ (eq)	BnOH (eq)	DBU (eq)	BnBr (eq)	Yield (%)
1	2.5	2.0	7.6	2.0	25
2	2.9	2.0	8.8	2.0	26
3	3.3	1.5	10.0	1.5	22
4	3.3	1.5	10.0	3.0	23
5	3.3	2.0	10.0	2.0	32
6	3.3	2.0	10.0	5.0	33
7	3.3	3.0	10.0	3.0	42
8	3.3	5.0	10.0	5.0	45
9	3.7	2.0	11.2	2.0	23

^a^ All reactions were run in the 0.05 mmol scale of conjugate ketoester **2x** (1.0 eq, [c] = 0.12 M) and BnOH (2.0 eq) for formation of **8xh** in DME for 2 h at 40 °C; BnBr (2.0 eq) for 2 h at 25 °C for the formation of **1xh**.

**Table 3:** Synthesis of derivatives of 1-alkoxyindole **1** ^a^.

Entry	ROH	RX	Product	Yield (%)
1	MeOH	MeI	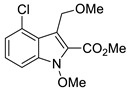 **1xa**	43
2	EtOH	EtBr	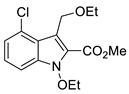 **1xb**	31
3	*n*-PrOH	*n*-PrBr	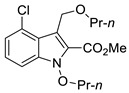 **1xc**	30
4	*n*-BuOH	*n*-BuBr	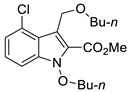 **1xd**	32
5	*n*-PenOH	*n*-PenBr	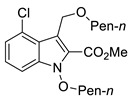 **1xe**	20
6	*n*-HexOH	*n*-HexBr	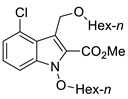 **1xf**	21
7	*n*-OctOH	*n*-OctBr	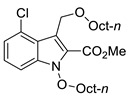 **1xg**	22
8	BnOH	BnBr	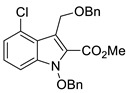 **1xh**	32
9	PhCH_2_CH_2_OH	PhCH_2_CH_2_Br	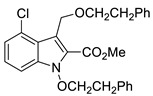 **1xi**	18
10	*i*-PrOH	*i*-PrBr	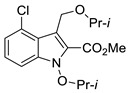 **1xj**	12
11	*c*-HexOH	*c*-HexBr	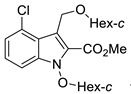 **1xk**	11
12	PhCH_2_CH_2_OH	MeI	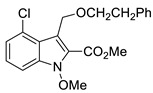 **1xl**	22
13	MeOH	*n*-OctBr	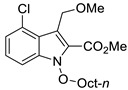 **1xm**	33
14	*n*-OctOH	MeI	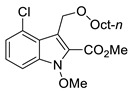 **1xn**	36
15	MeOH	MeI	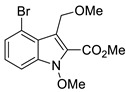 **1ya**	52
16	EtOH	EtBr	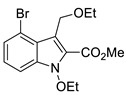 **1yb**	26
17	*n*-PrOH	*n*-PrBr	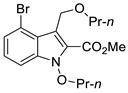 **1yc**	28
18	*n*-OctOH	*n*-OctBr	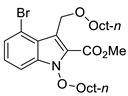 **1yg**	21
19	BnOH	BnBr	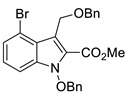 **1yh**	41
20	*i*-PrOH	*i*-PrBr	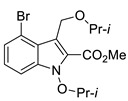 **1yj**	16
21	PhCH_2_CH_2_OH	MeI	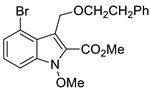 **1yl**	25
22	PhCH_2_CH_2_OH	*n*-PenBr	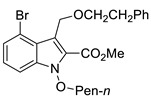 **1ym**	21

^a^ Reactions were run in 0.05–0.22 mmol scale of conjugate ketoester **2** (1.0 eq) and R^1^OH (2.0 eq) for formation of **8** in DME for 1–2 h at 40 °C; R^2^Y (2.0 eq) for 1–4 h at 25–50 °C for formation of **1**.

## Data Availability

The data presented in this study are available in insert article or supplementary material here.

## Supplementary Materials

The charts for ^1^H- and ^13^C-NMR spectroscopies are available online.

## Sample Availability

Samples of the compounds **1xa**–**1ym** are available from the authors.

## References

[1] Somei M. (2002). Recent advances in the chemistry of 1-hydroxytryptophans, and 1-hydroxytryptamines. Adv. Heterocycl. Chem..

[2] Escolano C. (2005). Stephacidin B, the avrainvillamide dimer: A formidable synthetic challenge. Angew. Chem. Int. Ed..

[3] Wang J., Pearce A.N., Chan S.T.S., Taylor R.B., Page M.J., Valentine A., Bourguet-Kondracki M.-L., Dalton J.P., Wiles S., Copp B.R. (2016). Biologically active acetylenic amino alcohol and *N*-hydroxylated 1,2,3,4-tetrahydro-β-carboline constituents of the New Zealand ascidian *pseudodistoma opacum*. J. Nat. Prod..

[4] Kinoshita T., Tatara S., Ho F.-C., Sankawa U. (1989). 3-Prenylindoles from *murraya paniculata* and their biogenetic significance. Phytochemistry.

[5] Bagley M.C., Dale J.W., Merritt E.A., Xiong X. (2005). Thiopeptide antibiotics. Chem. Rev..

[6] Granchi C., Roy S., Giacomelli C., Macchia M., Tuccinardi T., Martinelli A., Lanza M., Betti L., Giannaccini G., Lucacchini A. (2011). Discovery of *N*-hydroxyindole-based inhibitors of human lactate dehydrogenase isoform A (LDH-A) as starvation agents against cancer cells. J. Med. Chem..

[7] Jump S.M., Kung J., Staub R., Kinseth M.A., Cram E.J., Yudina L.N., Preobrazhenskaya M.N., Bjeldanes L.F., Firestone G.L. (2008). *N*-Alkoxy derivatization of indole-3-carbinol increases the efficacy of the G1 cell cycle arrest and of I3C-specific regulation of cell cycle gene transcription and activity in human breast cancer cells. Biochem. Pharmacol..

[8] Acheson R.M. (1990). 1-Hydroxypyrroles, 1-hydroxyindoles and 9-hydroxycarbazoles. Adv. Heterocycl. Chem..

[9] Nicolaou K.C., Lee S.H., Estrada A.A., Zak M. (2005). Construction of substituted *N*-hydroxyindoles: Synthesis of a Nocathiacin Ⅰ model system. Angew. Chem, Int. Ed..

[10] Park Y.K., Kim H., Kim D.S., Cho H., Moon A., Jeong C., Yoon H.-R., Lee S.H. (2015). Synthesis of new 2,3-disubstituted 4-chloro-1-hydroxyindoles. Bull. Kor. Chem. Soc..

[11] Lee S.H., Kim H., Park Y.K., Cho H. (2015). Synthetic of new 3-[(alkylthio)methyl]-1-hydroxy-2-phenylindoles. Synlett.

[12] Kim H., Lee S.H. (2016). Synthesis of new 3-substituted 1-hydroxy-2-phenylindoles using sulfur-containing nucleophiles. Heterocycles.

[13] Cho H., Kim H., Lim Y.J., Lee S.H. (2018). Synthesis of new 3-[(alkylthio)methyl]-1-hydroxy-2-(4′-substituted phenyl)indoles and their mechanistic studies on substituent effects. Arkivoc.

[14] Somei M., Kawasaki T. (1989). A new and simple synthesis of 1-hydroxyindole derivatives. Heterocycles.

[15] Yun Z., Cheng R., Sun J., Zhang-Negrerie D., Du Y. (2018). Iodobenzene dichloride/zinc chloride-mediated synthesis of *N*-alkoxyindole-3-carbonitriles from 3-alkoxyimino-2-arylalkylnitriles via intramolecular heterocyclization. Adv. Synth. Catal..

[16] Somei M. (1999). 1-Hydroxyindoles. Heterocycles.

[17] Nicolaou K.C., Estrada A.A., Lee S.H., Freestone G.C. (2006). Synthesis of highly substituted *N*-hydroxyindoles through 1,5-addition of carbon nucleophiles to in situ generated unsaturated nitrones. Angew. Chem. Int. Ed..

[18] Bellamy F.D., Ou K. (1984). Selective reduction of aromatic nitro compounds with stannous chloride in non acidic and non aqueous medium. Tetrahedron Lett..

[19] Park Y.K., Lee S.H. (2017). Synthesis of new 1-hydroxyindole-2-carboxylates and mechanistic studies on reaction pathways. J. Heterocyclic Chem..

[20] Kolthoff I.M., Chantooni J.M.K., Bhowmik S. (1986). Dissociation constant of uncharged and monovalent cation acids in dimethyl sulfoxide. J. Am. Chem. Soc..

[21] Streitwieser A., Kim Y.J. (2000). Ion pair basicity of some amines in THF: Implications for ion pair acidity scales. J. Am. Chem. Soc..

