# Supplementary material for: One-Pot Synthesis of Novel Multisubstituted 1-Alkoxyindoles
Source: Molecules. 2021 Mar 8;26(5):1466. doi: 10.3390/molecules26051466 (PMC7962848; doi:10.3390/molecules26051466)
Supplement: Supplementary file 1 [file molecules-26-01466-s001.pdf]

Supplementary Materials

for

One-pot Synthesis of Novel Multisubstituted 1-Alkoxyindoles

Ye Eun Kim, Hyunsung Cho, Yoo Jin Lim, Chorong Kim, and Sang Hyup Lee*

College of Pharmacy and Innovative Drug Center, Duksung Women's University,

Seoul 01369, Republic of Korea,

*sanghyup@duksung.ac.kr

^{1}H and ^{13}C NMR spectrum of compound **4x** --------------------------------- S3

^{1}H and ^{13}C NMR spectrum of compound **4y** --------------------------------- S4

^{1}H and ^{13}C NMR spectrum of compound **2x** --------------------------------- S5

^{1}H and ^{13}C NMR spectrum of compound **2y** --------------------------------- S6

^{1}H and ^{13}C NMR spectrum of compound **1xa** -------------------------------- S7

^{1}H and ^{13}C NMR spectrum of compound **1xb** -------------------------------- S8

^{1}H and ^{13}C NMR spectrum of compound **1xc** -------------------------------- S9

^{1}H and ^{13}C NMR spectrum of compound **1xd** -------------------------------- S10

^{1}H and ^{13}C NMR spectrum of compound **1xe** -------------------------------- S11

^{1}H and ^{13}C NMR spectrum of compound **1xf** --------------------------------- S12

^{1}H and ^{13}C NMR spectrum of compound **1xg** -------------------------------- S13

^{1}H and ^{13}C NMR spectrum of compound **1xh** -------------------------------- S14

^{1}H and ^{13}C NMR spectrum of compound **1xi** --------------------------------- S15

^{1}H and ^{13}C NMR spectrum of compound **1xj** --------------------------------- S16

^{1}H and ^{13}C NMR spectrum of compound **1xk** -------------------------------- S17

^{1}H and ^{13}C NMR spectrum of compound **1xl** --------------------------------- S18

^{1}H and ^{13}C NMR spectrum of compound **1xm** ------------------------------- S19

^{1}H and ^{13}C NMR spectrum of compound **1xn** -------------------------------- S20

^{1}H and ^{13}C NMR spectrum of compound **1ya** -------------------------------- S21

^{1}H and ^{13}C NMR spectrum of compound **1yb** -------------------------------- S22

^{1}H and ^{13}C NMR spectrum of compound **1yc** -------------------------------- S23

^{1}H and ^{13}C NMR spectrum of compound **1yg** -------------------------------- S24

^{1}H and ^{13}C NMR spectrum of compound **1yh** -------------------------------- S25

^{1}H and ^{13}C NMR spectrum of compound **1yj** --------------------------------- S26

^{1}H and ^{13}C NMR spectrum of compound **1yl** --------------------------------- S27

^{1}H and ^{13}C NMR spectrum of compound **1ym** ------------------------------- S28

^{1}H and ^{13}C NMR spectrum of compound **11xl** -------------------------------- S29

^{1}H and ^{13}C NMR spectrum of compound **11xm** ------------------------------ S30

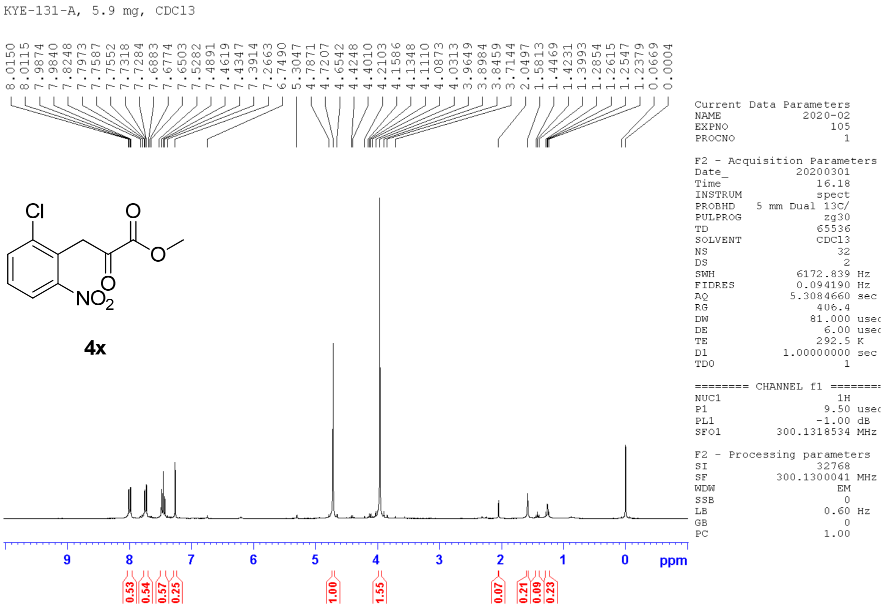

^{1}H NMR spectrum (300 MHz, $CDCl_3$) of compound 4x

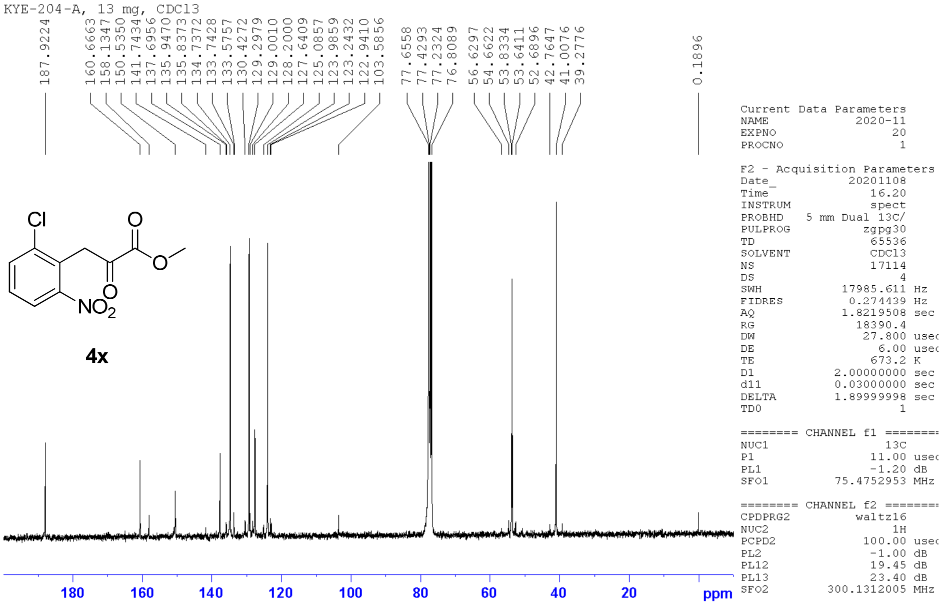

^{13}C NMR spectrum (75 MHz, $CDCl_3$) of compound 4x

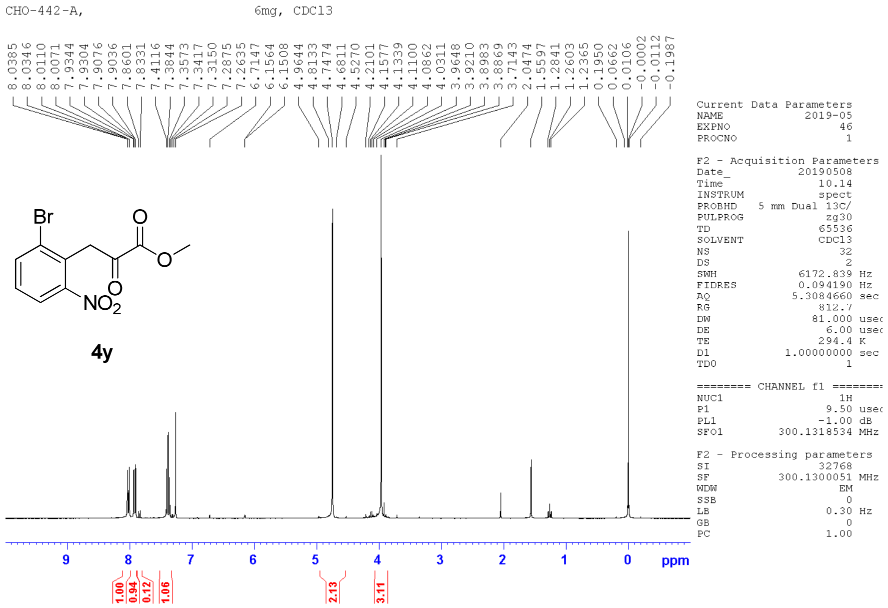

^{1}H NMR spectrum (300 MHz, $CDCl_3$) of compound 4y

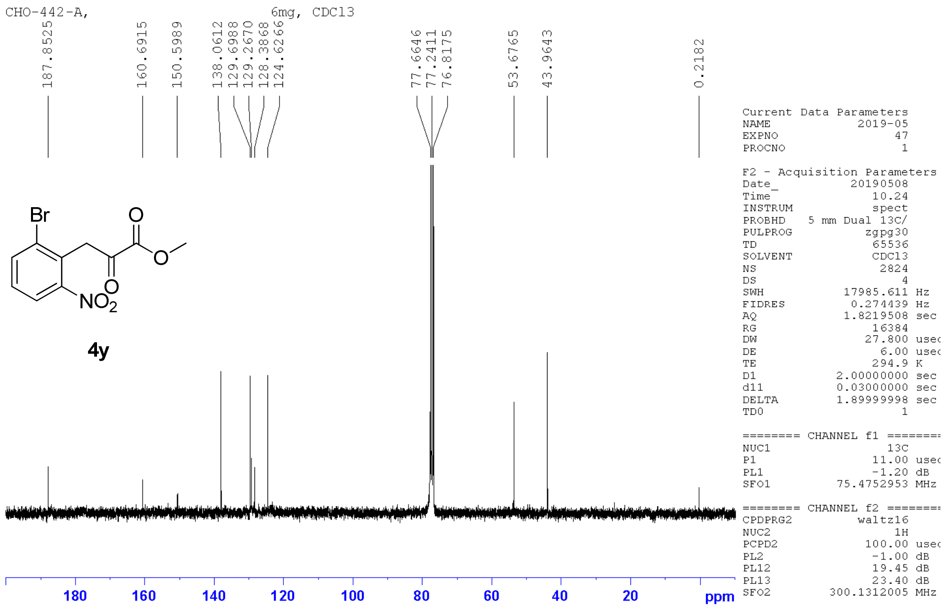

^{13}C NMR spectrum (75 MHz, $CDCl_3$) of compound 4y

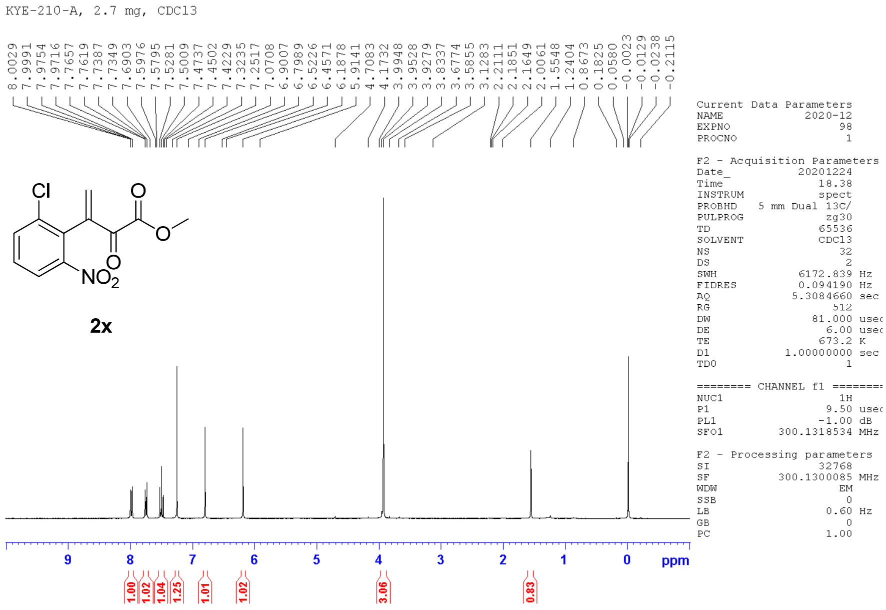

^{1}H NMR spectrum (300 MHz, CDCl3) of compound 2x

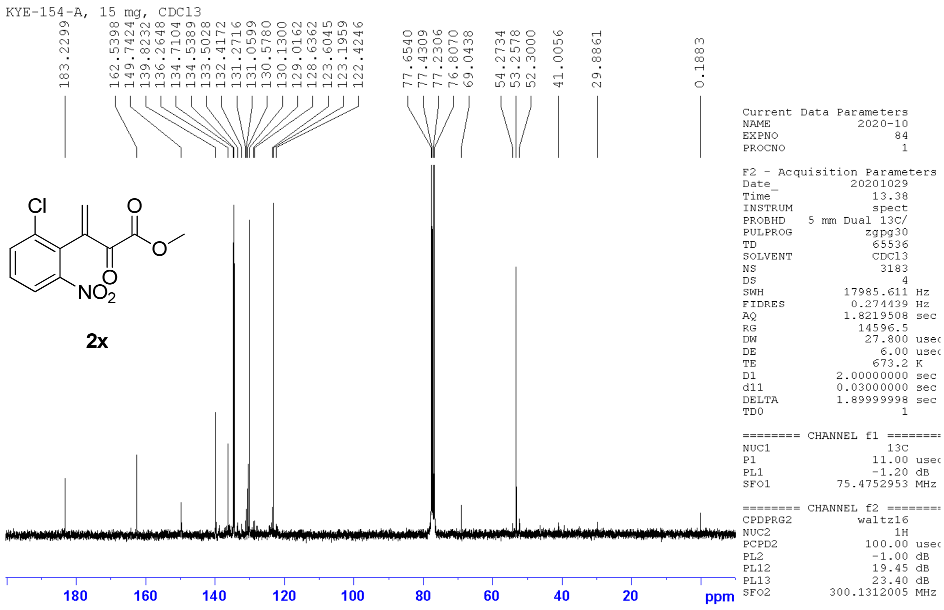

^{13}C NMR spectrum (75 MHz, CDCl3) of compound 2x

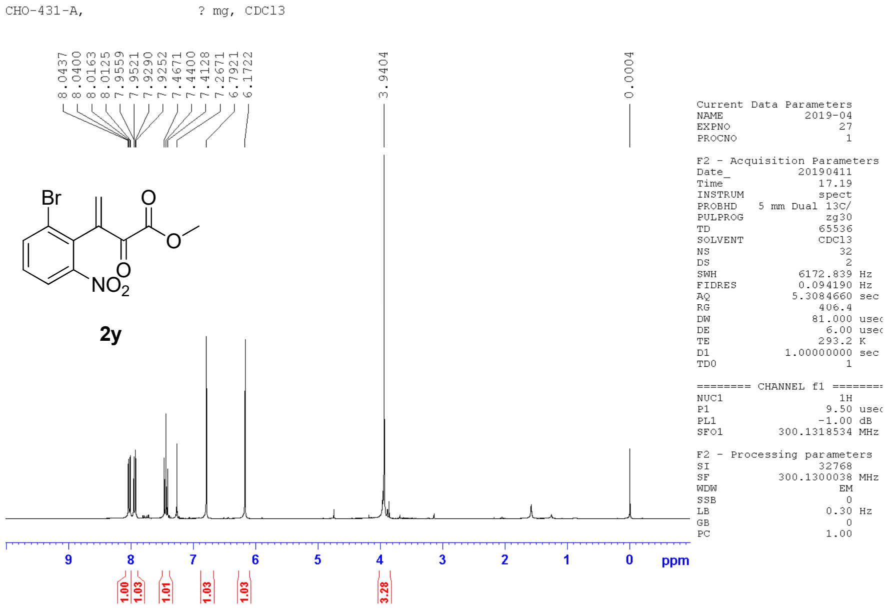

^{1}H NMR spectrum (300 MHz, $CDCl_3$) of compound 2y

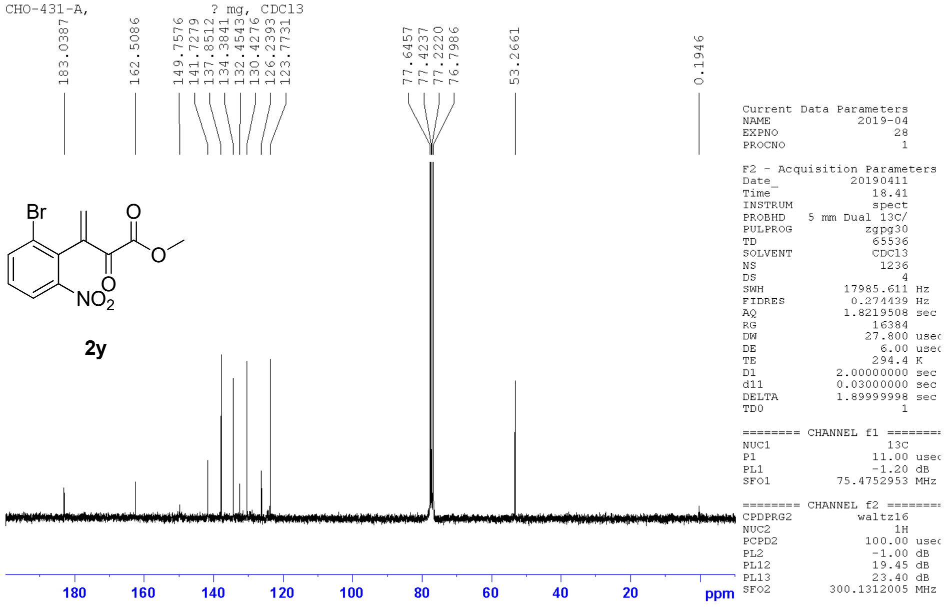

^{13}C NMR spectrum (75 MHz, $CDCl_3$) of compound 2y

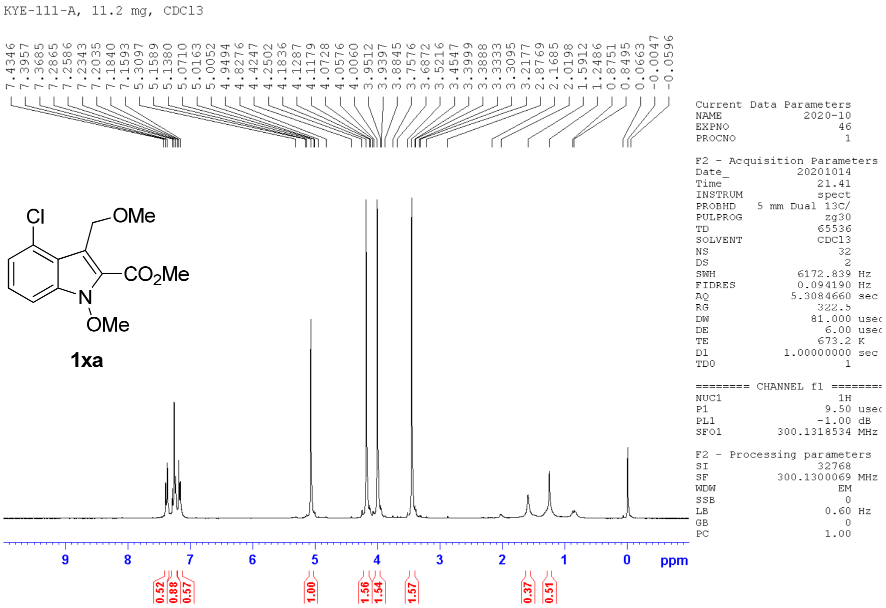

¹H NMR spectrum (300 MHz, CDCl₃) of compound 1xa

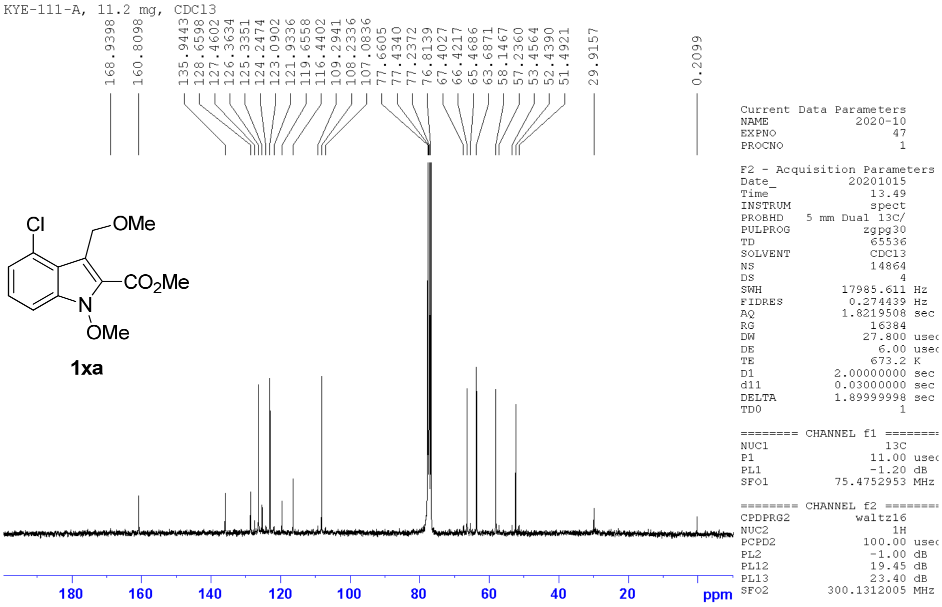

¹³C NMR spectrum (75 MHz, CDCl₃) of compound 1xa

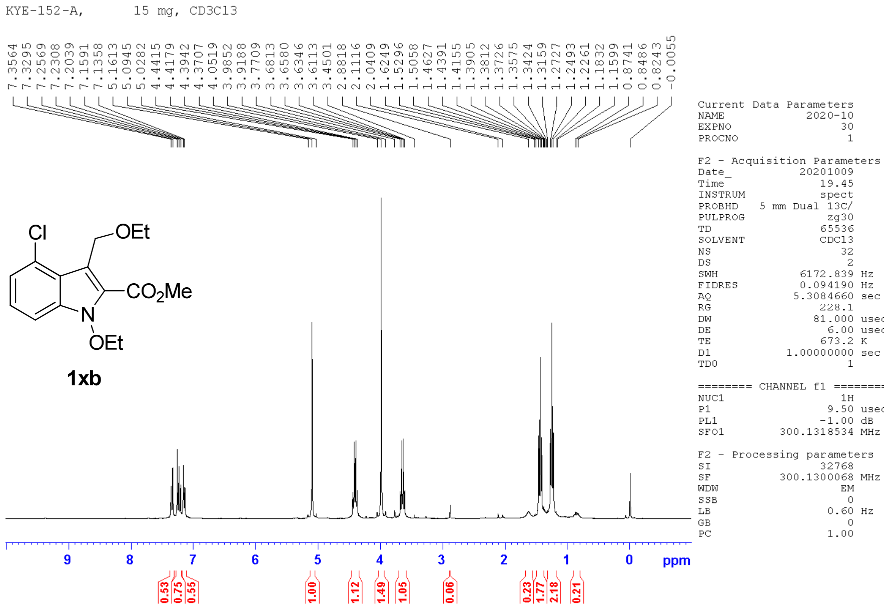

^{1}H NMR spectrum (300 MHz, $CDCl_3$) of compound 1xb

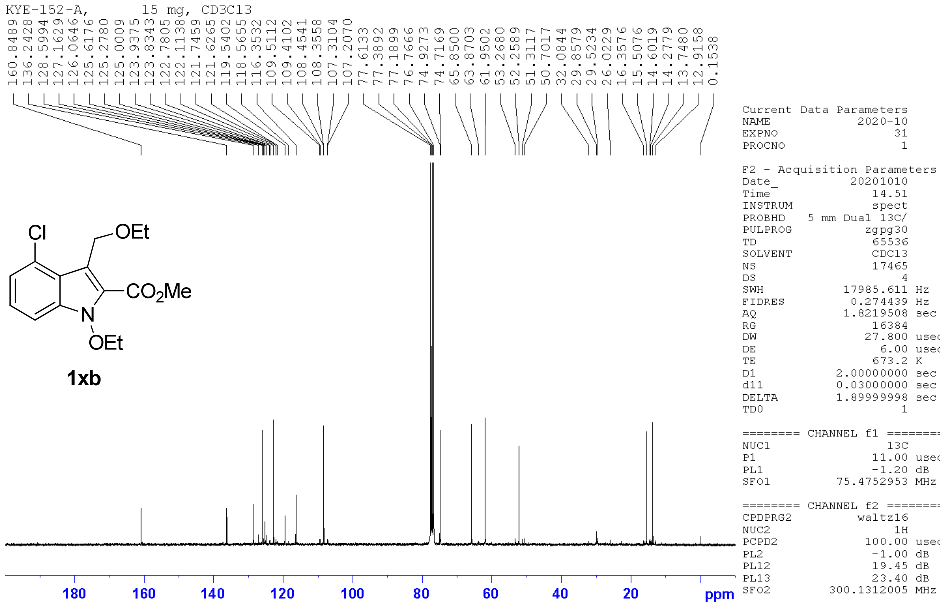

^{13}C NMR spectrum (75 MHz, $CDCl_3$) of compound 1xb

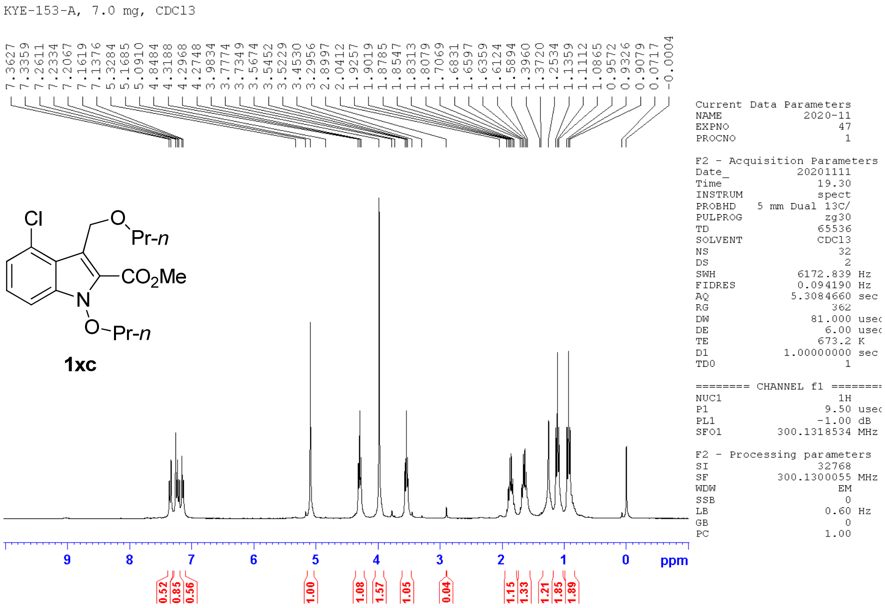

^{1}H NMR spectrum (300 MHz, $CDCl_3$) of compound 1xc

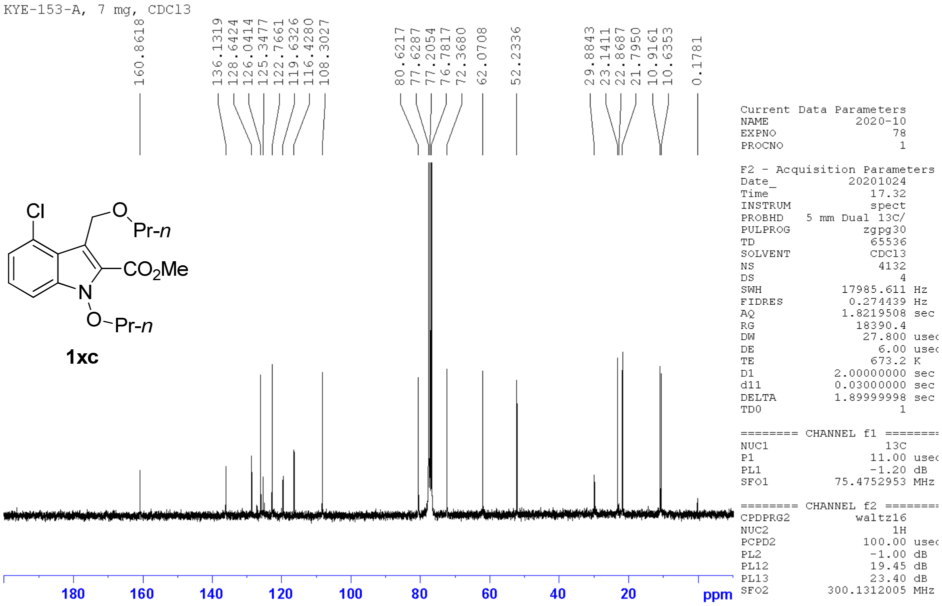

^{13}C NMR spectrum (75 MHz, $CDCl_3$) of compound 1xc

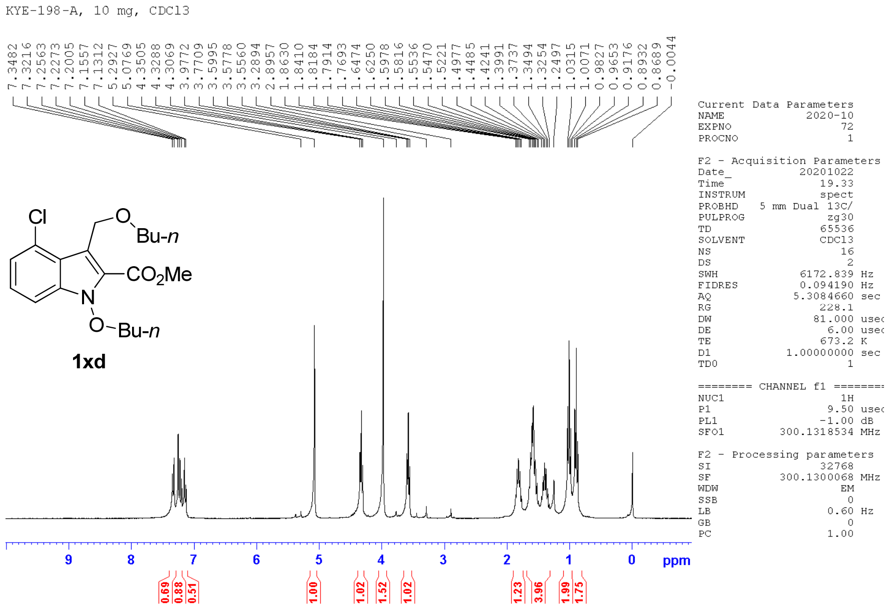

^{1}H NMR spectrum (300 MHz, $CDCl_3$) of compound 1xd

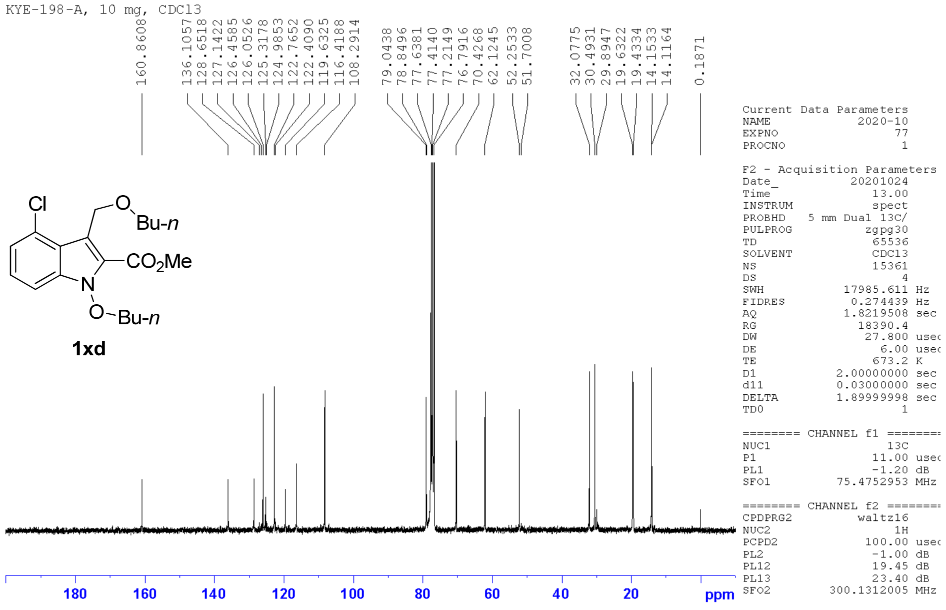

^{13}C NMR spectrum (75 MHz, $CDCl_3$) of compound 1xd

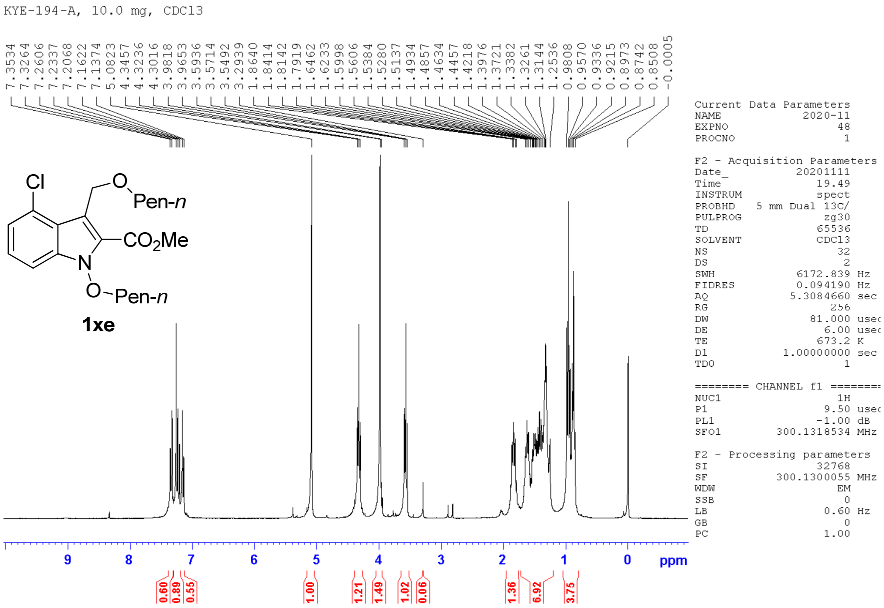

^{1}H NMR spectrum (300 MHz, CDCl3) of compound 1xe

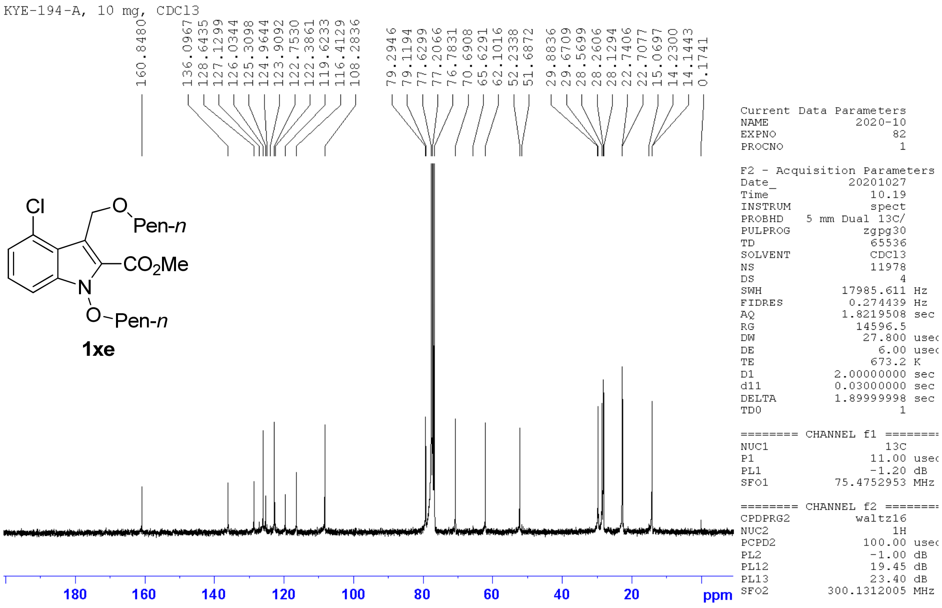

^{13}C NMR spectrum (75 MHz, CDCl3) of compound 1xe

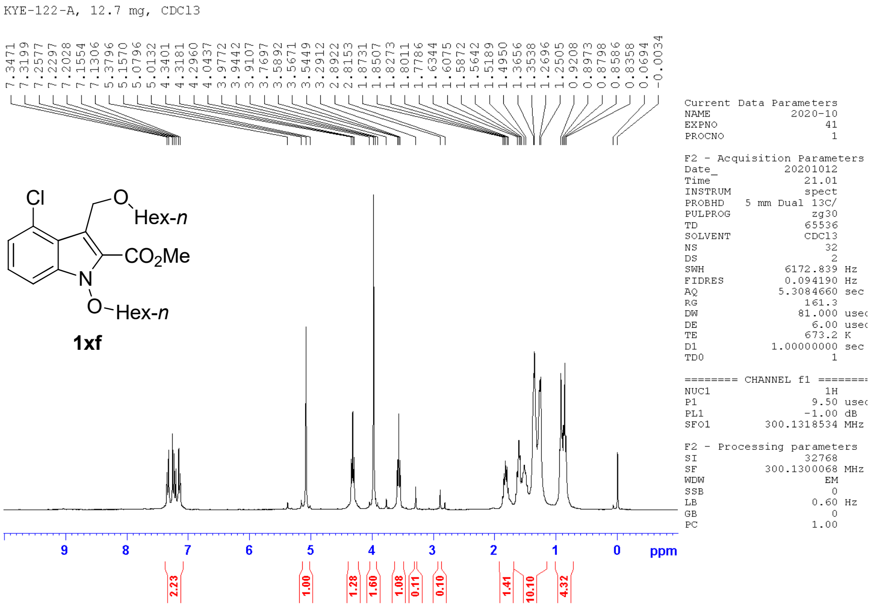

¹H NMR spectrum (300 MHz, CDCl₃) of compound 1xf

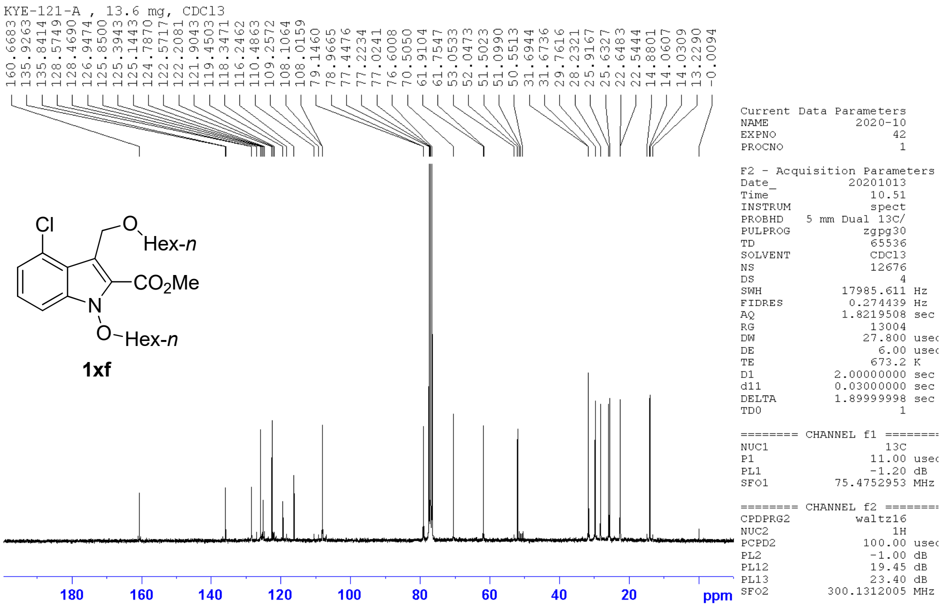

¹³C NMR spectrum (75 MHz, CDCl₃) of compound 1xf

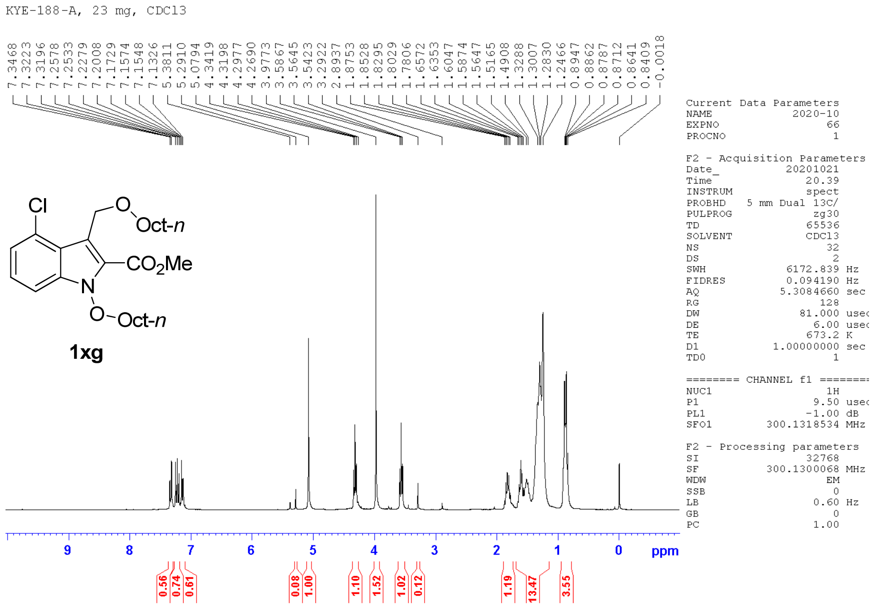

^{1}H NMR spectrum (300 MHz, $CDCl_3$) of compound 1xg

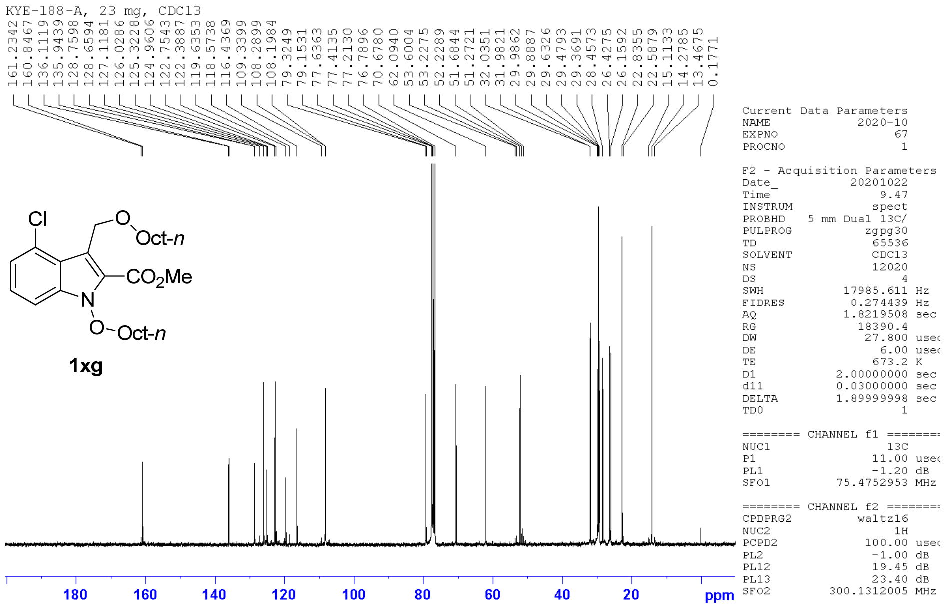

^{13}C NMR spectrum (75 MHz, $CDCl_3$) of compound 1xg

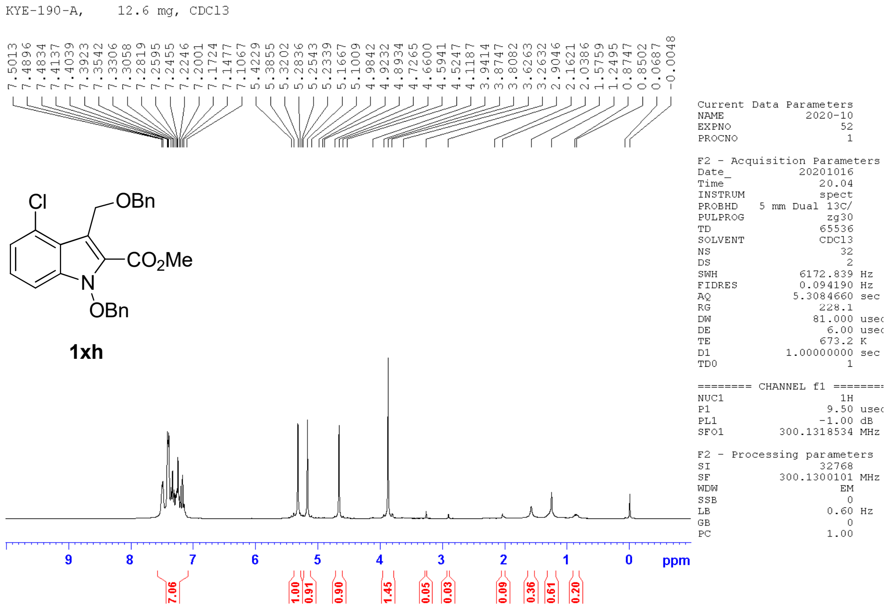

^{1}H NMR spectrum (300 MHz, CDCl$_3$) of compound 1xh

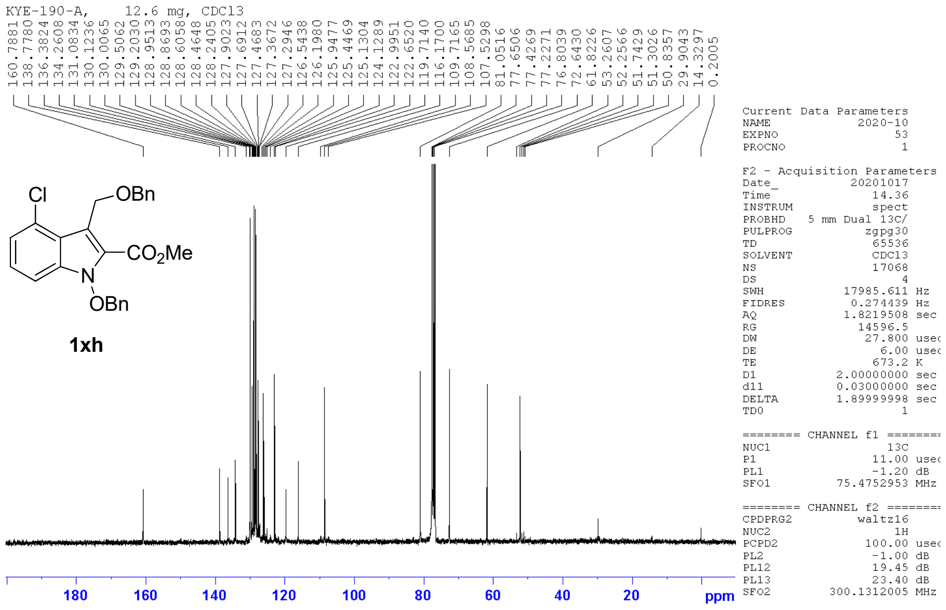

^{13}C NMR spectrum (75 MHz, CDCl$_3$) of compound 1xh

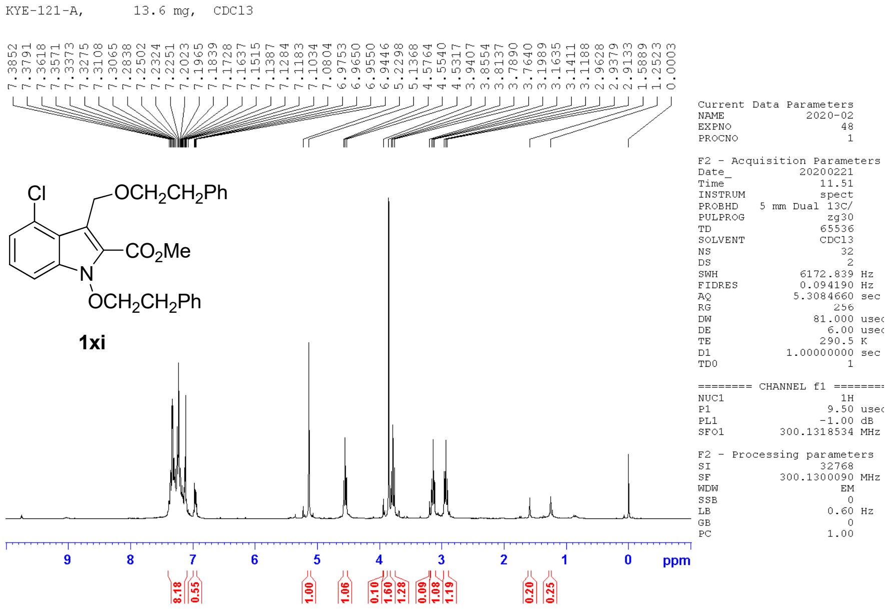

^{1}H NMR spectrum (300 MHz, $CDCl_3$) of compound 1xi

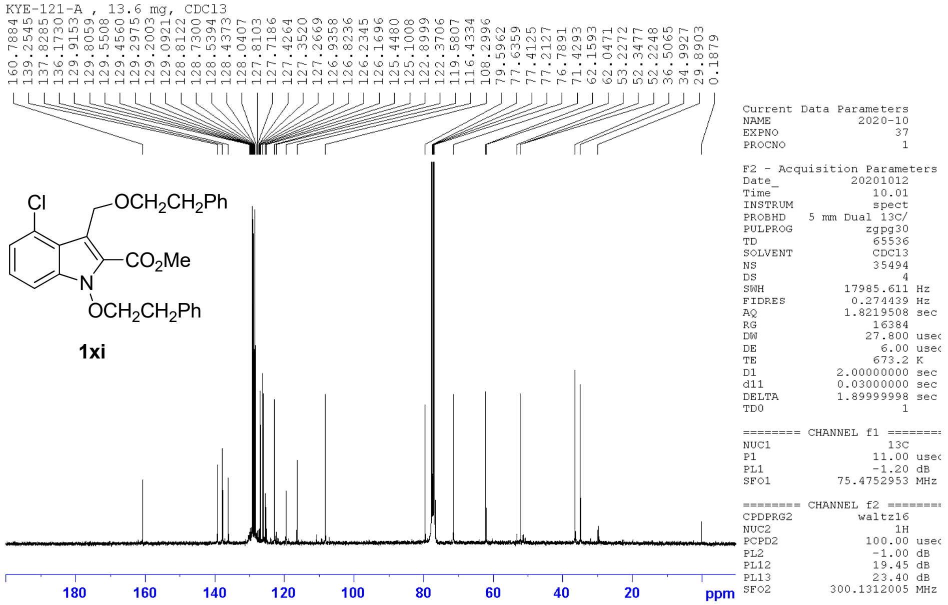

^{13}C NMR spectrum (75 MHz, $CDCl_3$) of compound 1xi

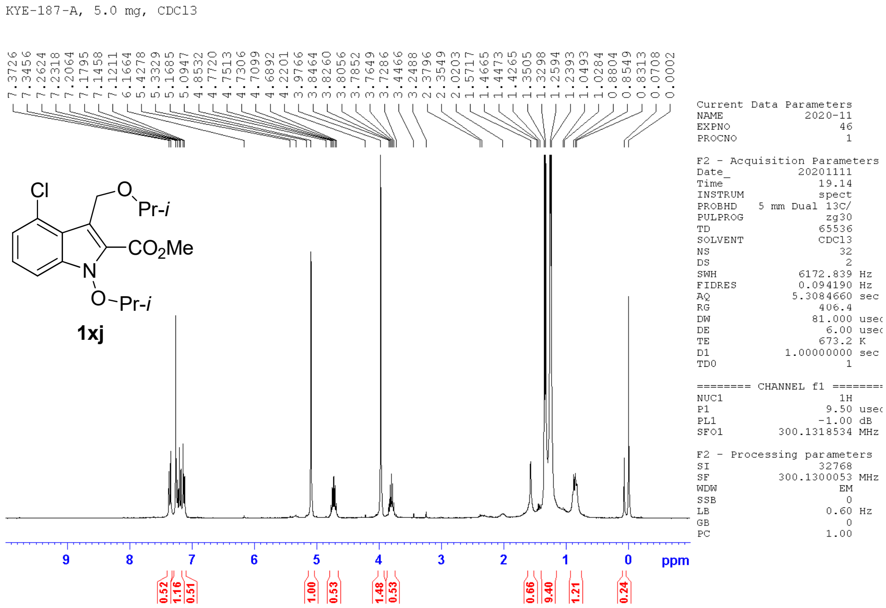

^{1}H NMR spectrum (300 MHz, CDCl3) of compound 1xj

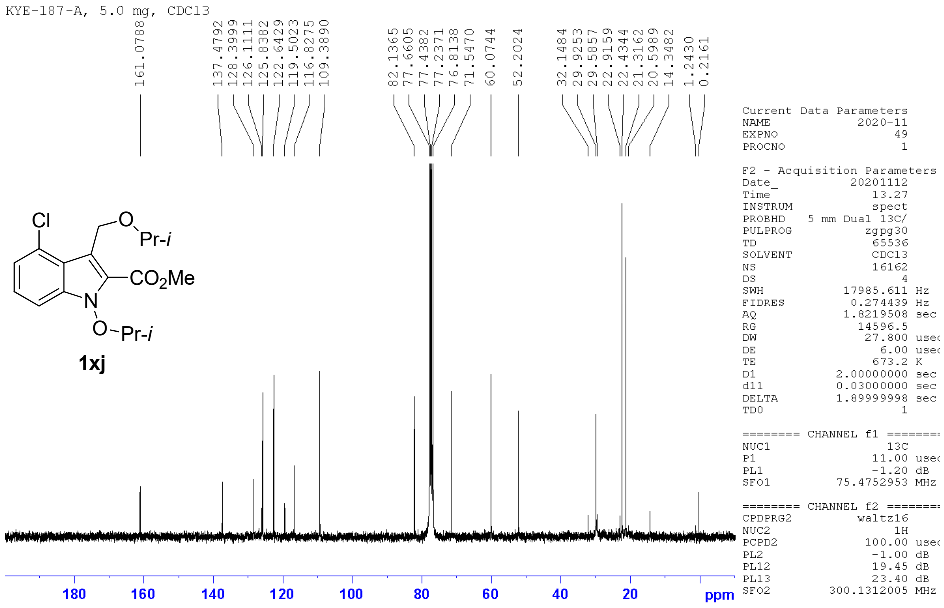

^{13}C NMR spectrum (75 MHz, CDCl3) of compound 1xj

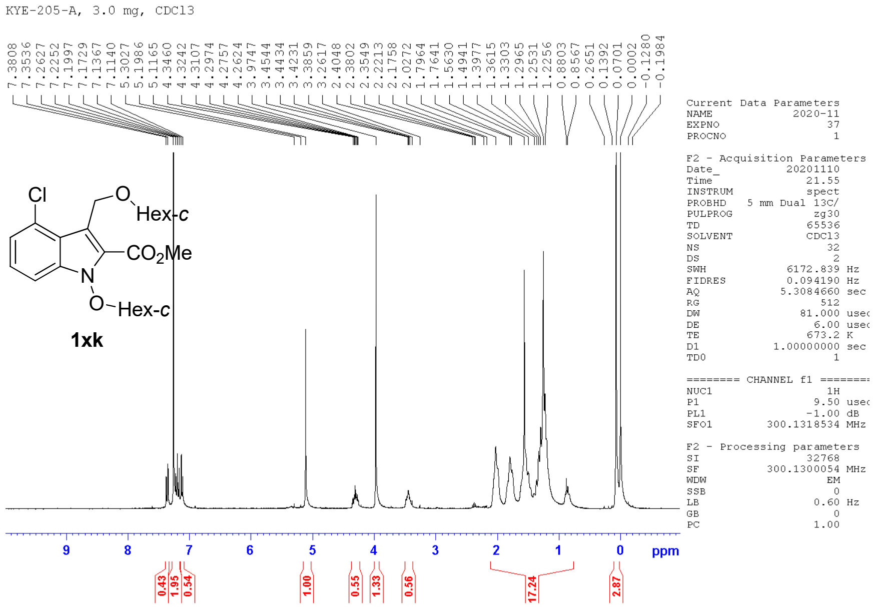

^{1}H NMR spectrum (300 MHz, $CDCl_3$) of compound 1xk

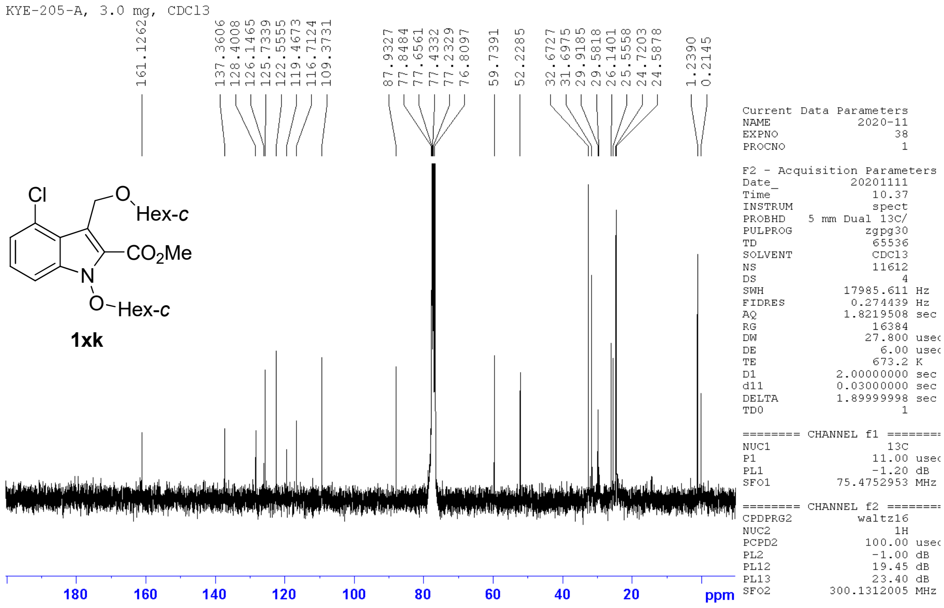

^{13}C NMR spectrum (75 MHz, $CDCl_3$) of compound 1xk

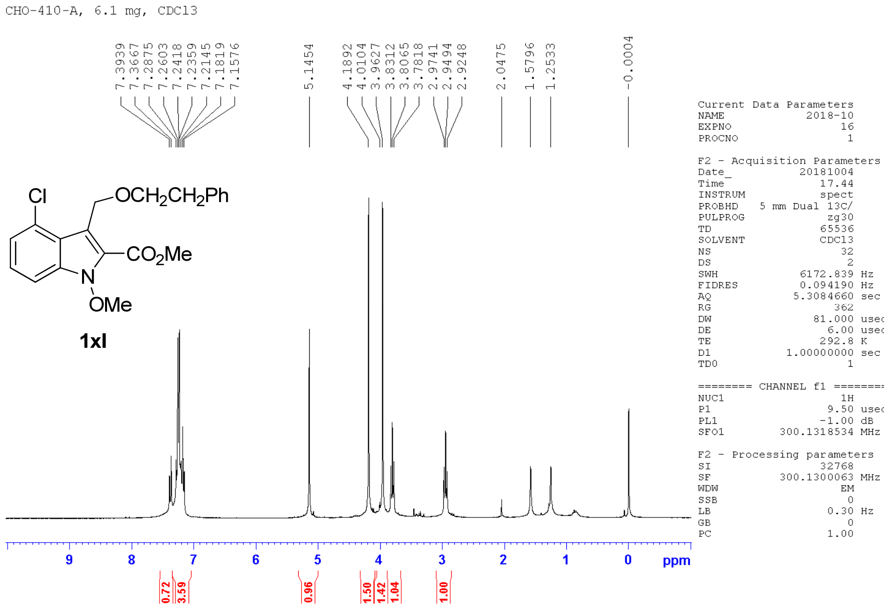

^{1}H NMR spectrum (300 MHz, $CDCl_3$) of compound 1xl

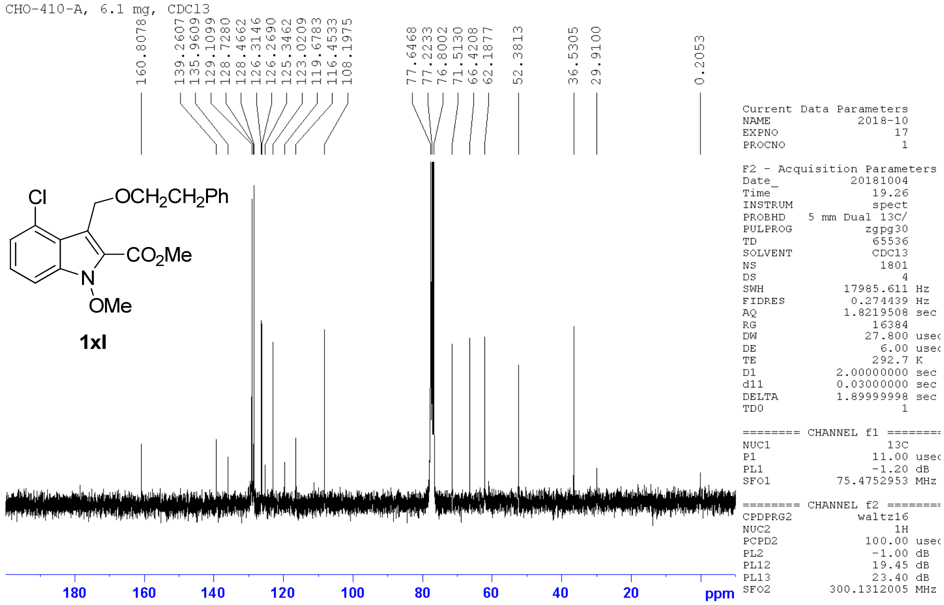

^{13}C NMR spectrum (75 MHz, $CDCl_3$) of compound 1xl

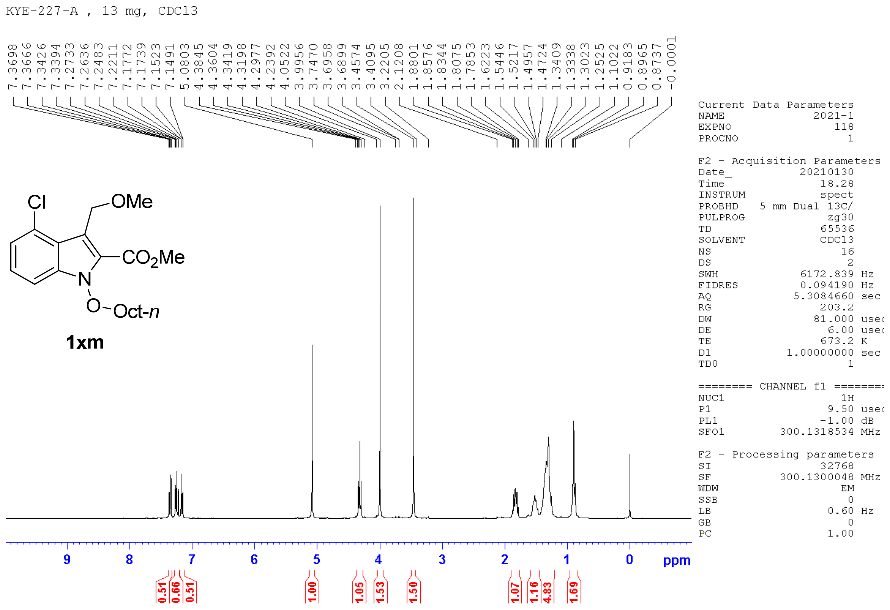

^{1}H NMR spectrum (300 MHz, $CDCl_3$) of compound 1xm

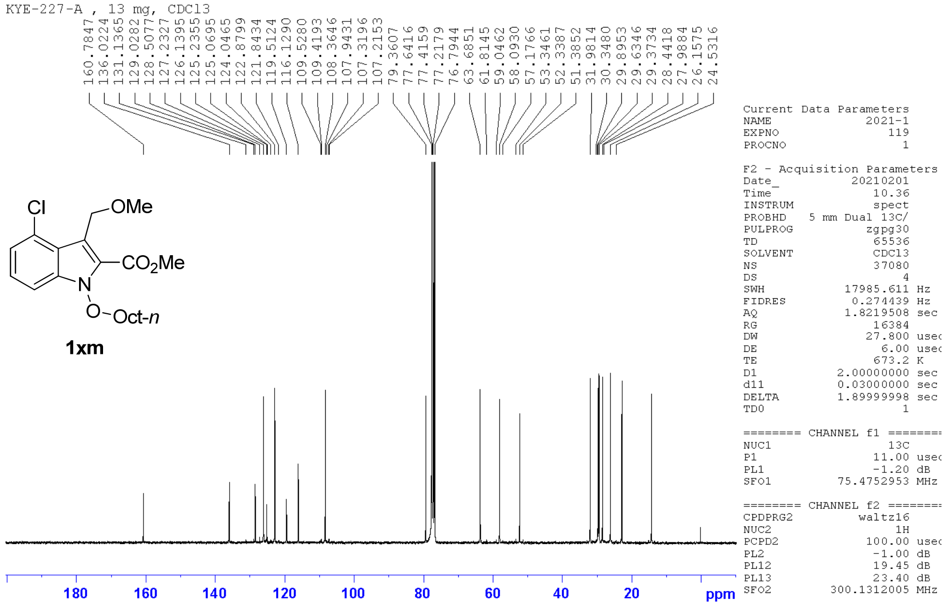

^{13}C NMR spectrum (75 MHz, $CDCl_3$) of compound 1xm

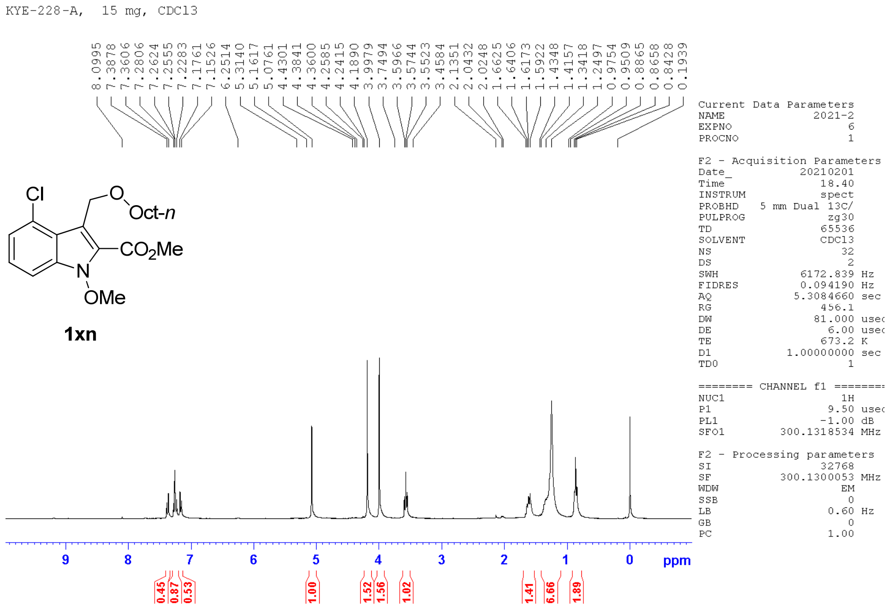

^{1}H NMR spectrum (300 MHz, $CDCl_3$) of compound 1xn

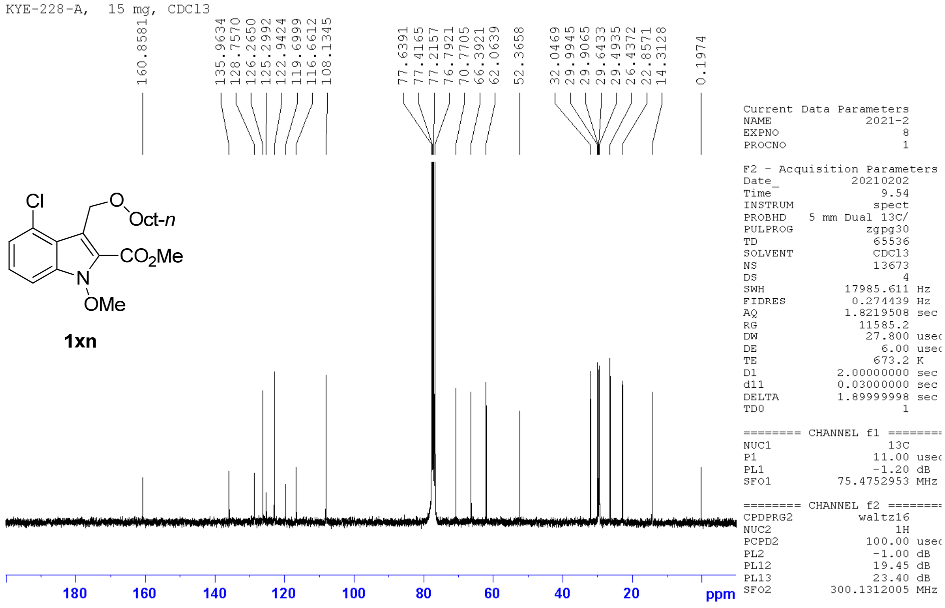

^{13}C NMR spectrum (75 MHz, $CDCl_3$) of compound 1xn

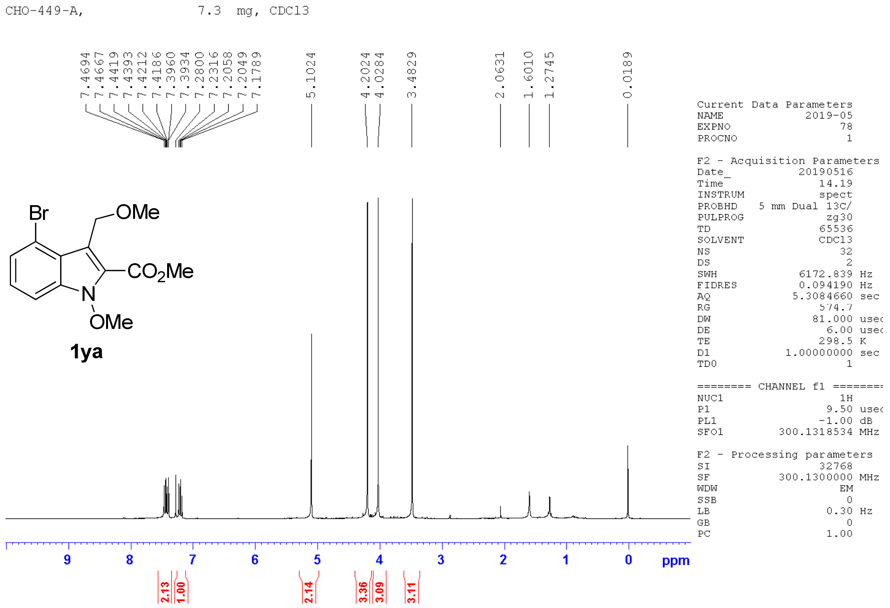

^{1}H NMR spectrum (300 MHz, $CDCl_3$) of compound 1ya

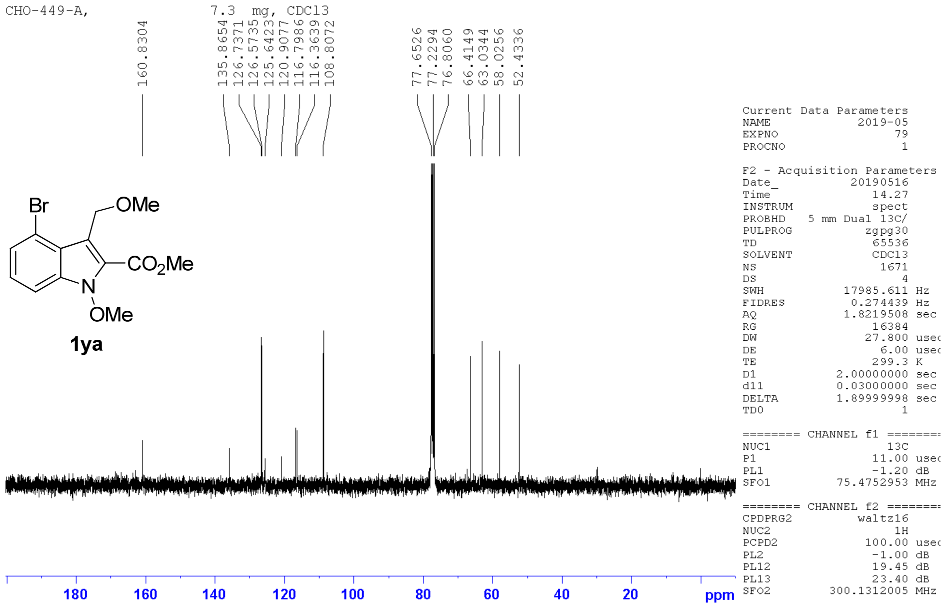

^{13}C NMR spectrum (75 MHz, $CDCl_3$) of compound 1ya

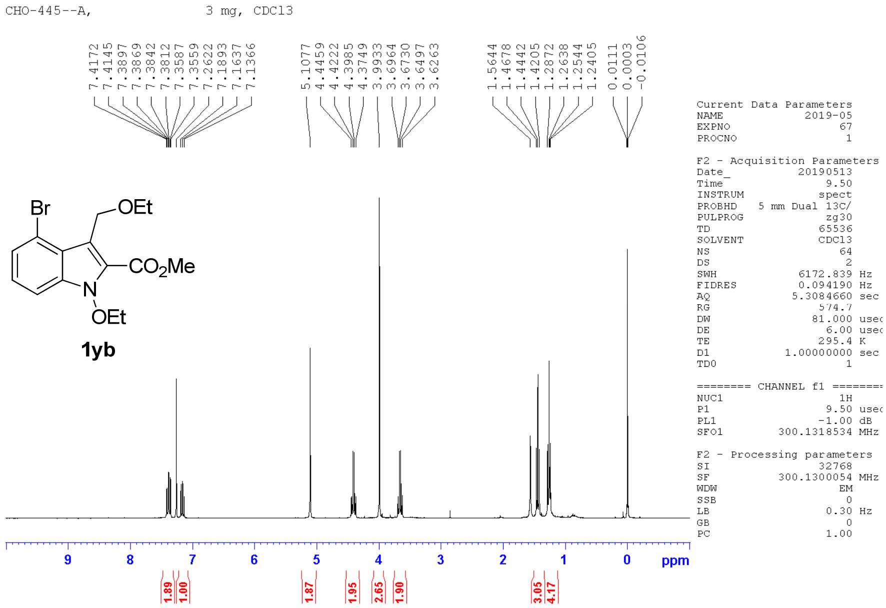

^{1}H NMR spectrum (300 MHz, $CDCl_3$) of compound 1yb

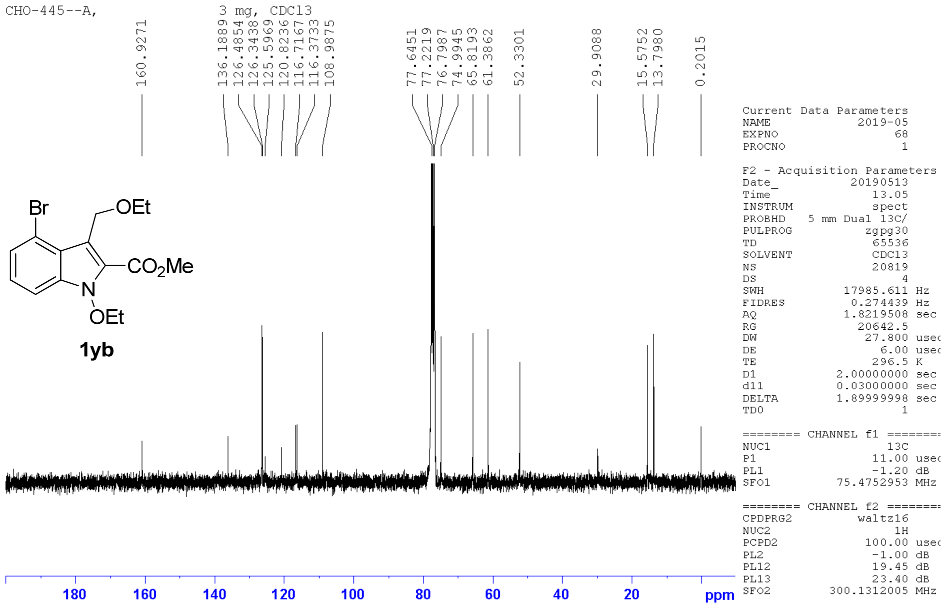

^{13}C NMR spectrum (75 MHz, $CDCl_3$) of compound 1yb

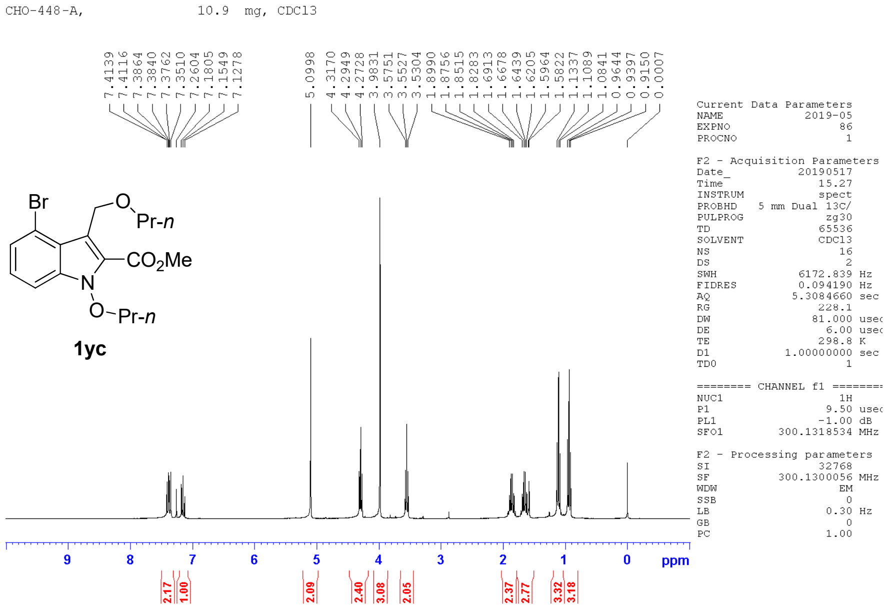

^{1}H NMR spectrum (300 MHz, $CDCl_3$) of compound 1yc

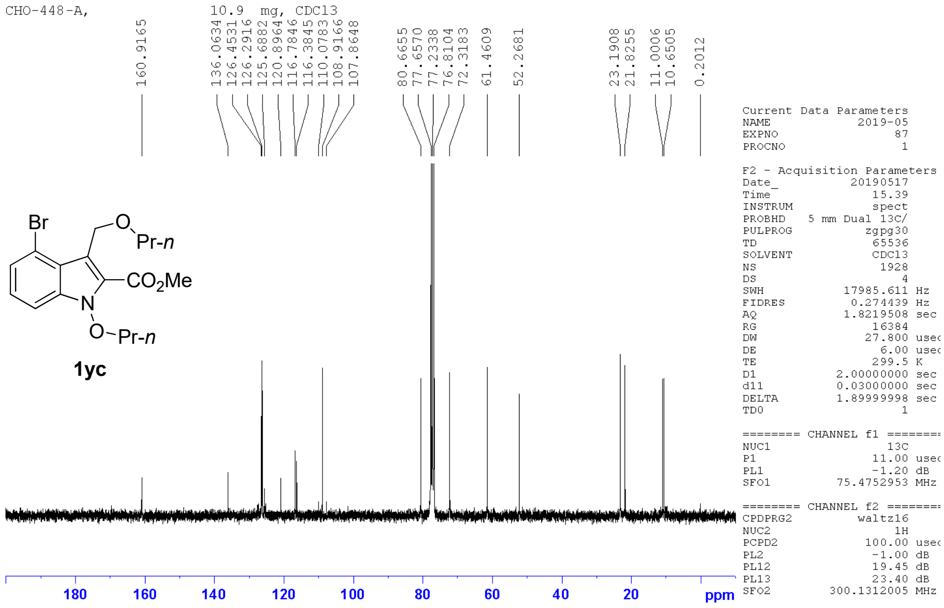

^{13}C NMR spectrum (75 MHz, $CDCl_3$) of compound 1yc

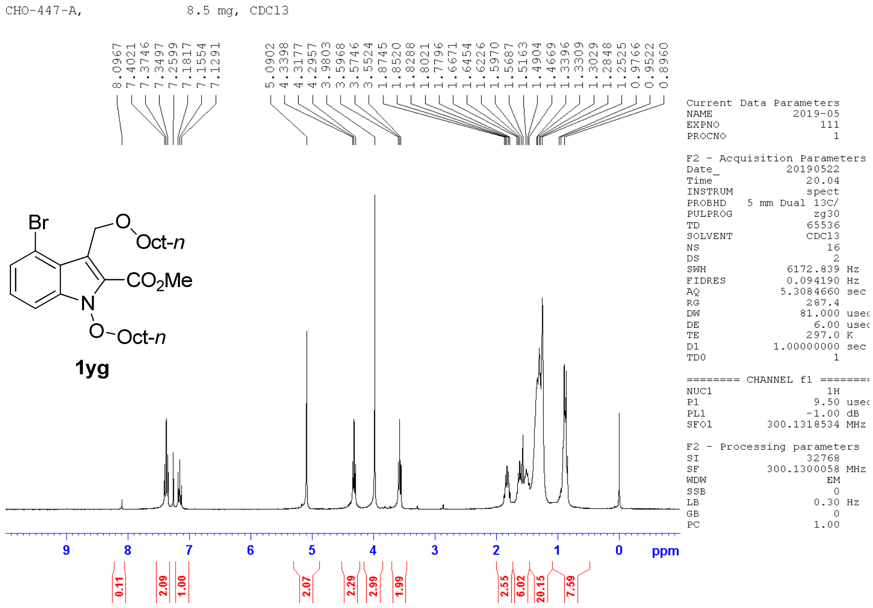

^{1}H NMR spectrum (300 MHz, $CDCl_3$) of compound 1yg

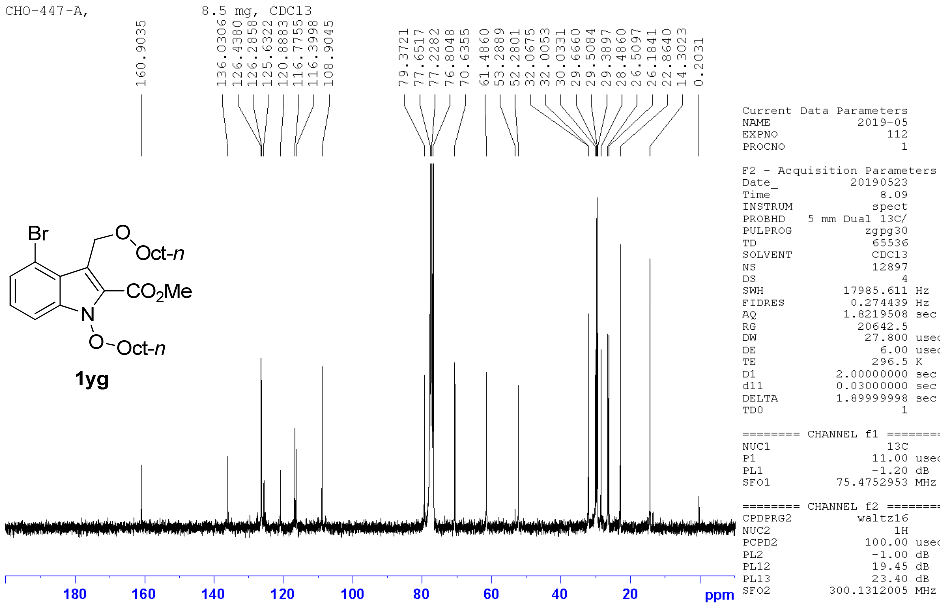

^{13}C NMR spectrum (75 MHz, $CDCl_3$) of compound 1yg

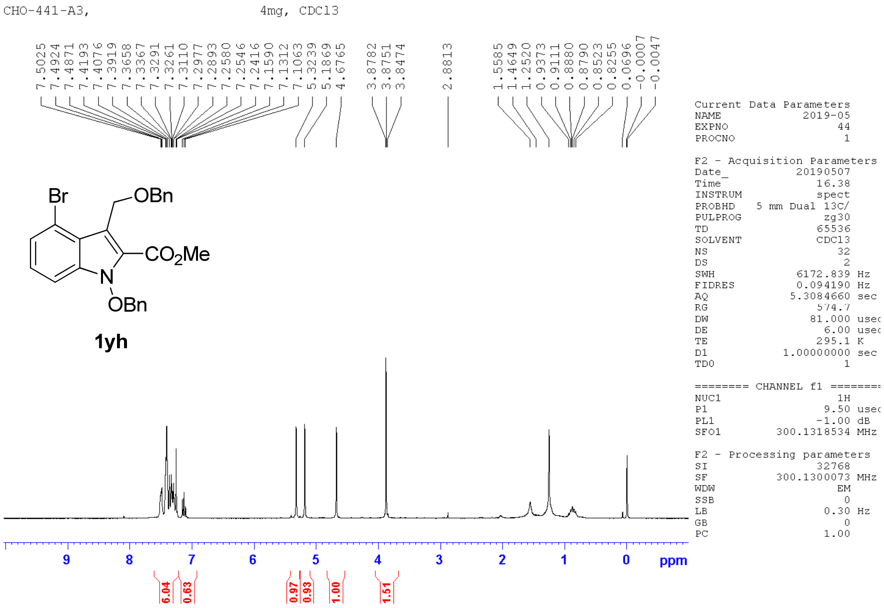

^{1}H NMR spectrum (300 MHz, $CDCl_3$) of compound 1yh

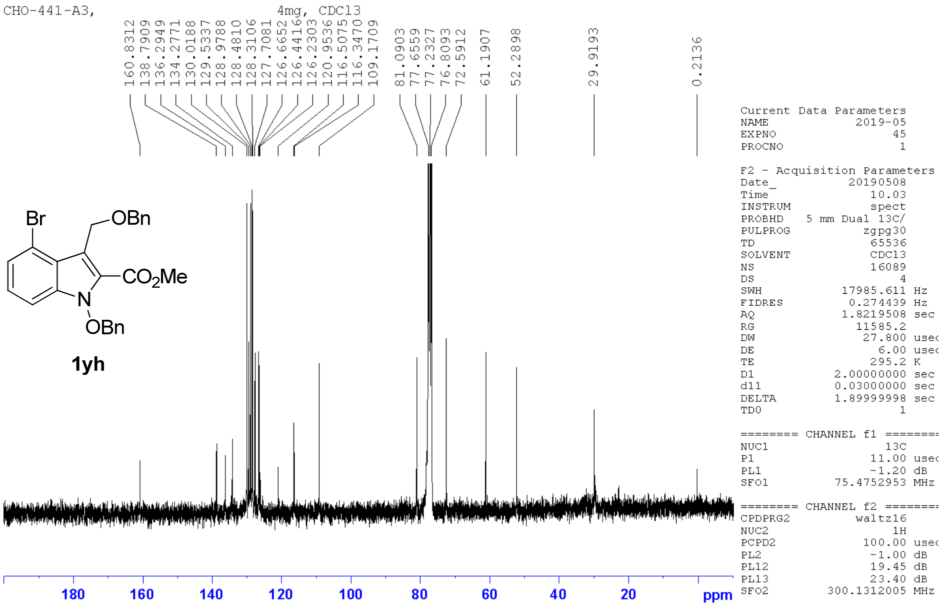

^{13}C NMR spectrum (75 MHz, $CDCl_3$) of compound 1yh

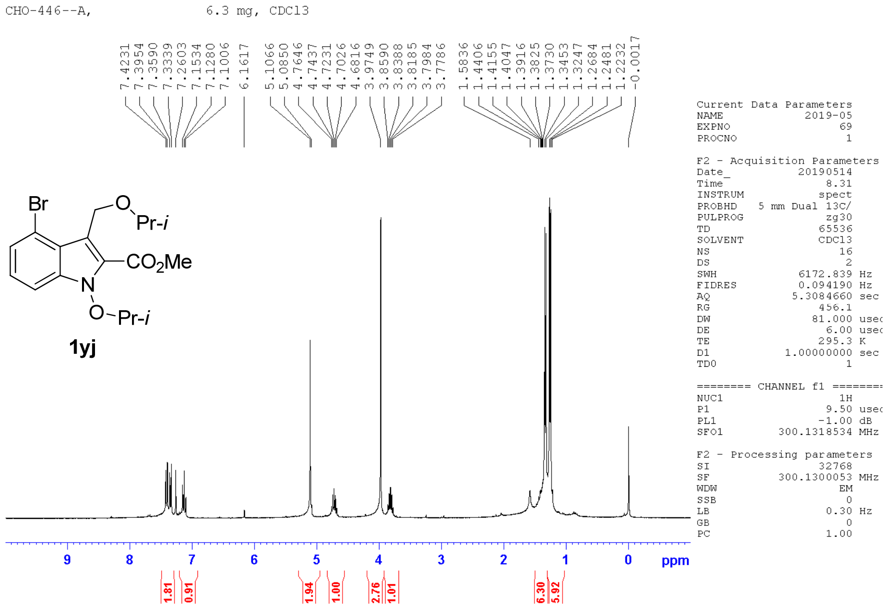

^{1}H NMR spectrum (300 MHz, CDCl$_3$) of compound 1yj

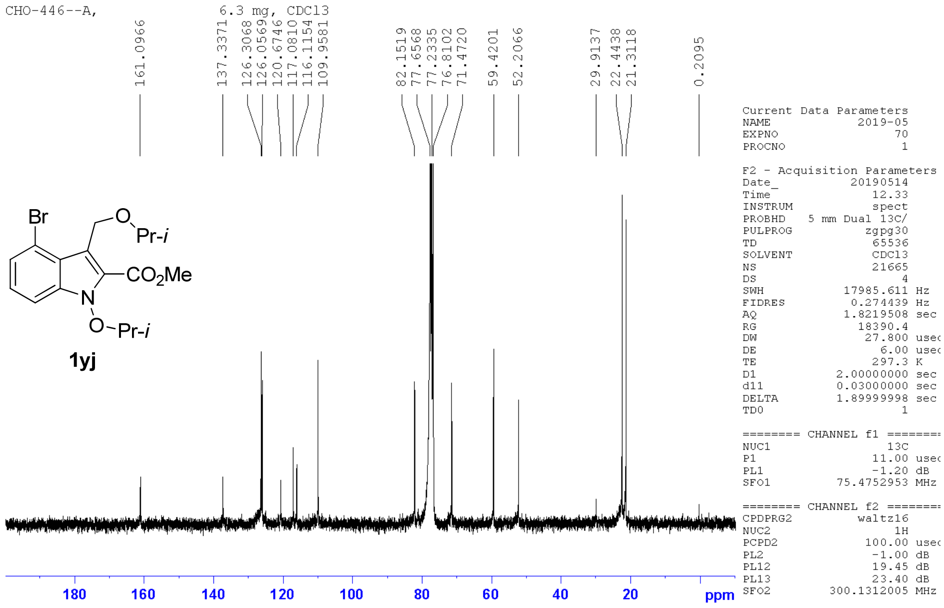

^{13}C NMR spectrum (75 MHz, CDCl$_3$) of compound 1yj

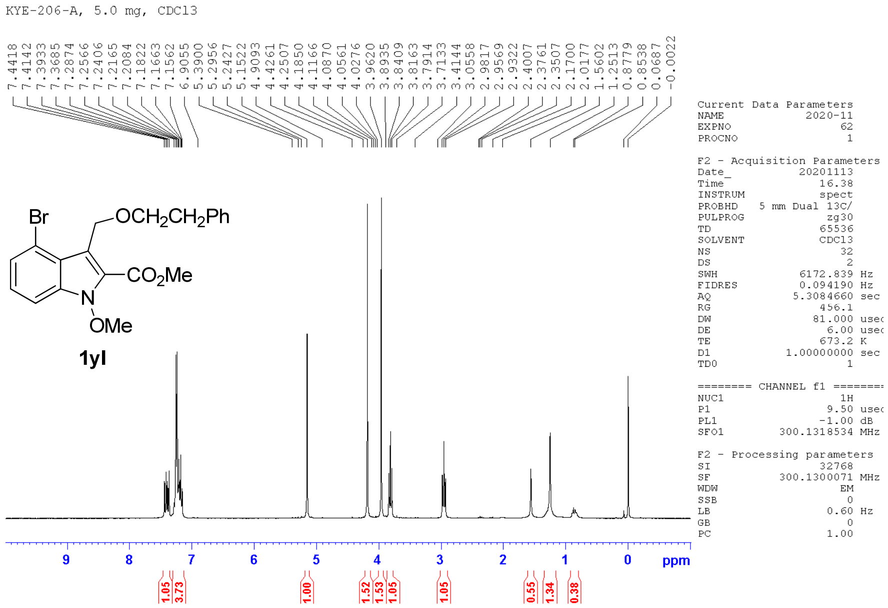

^{1}H NMR spectrum (300 MHz, CDCl$_3$) of compound 1yl

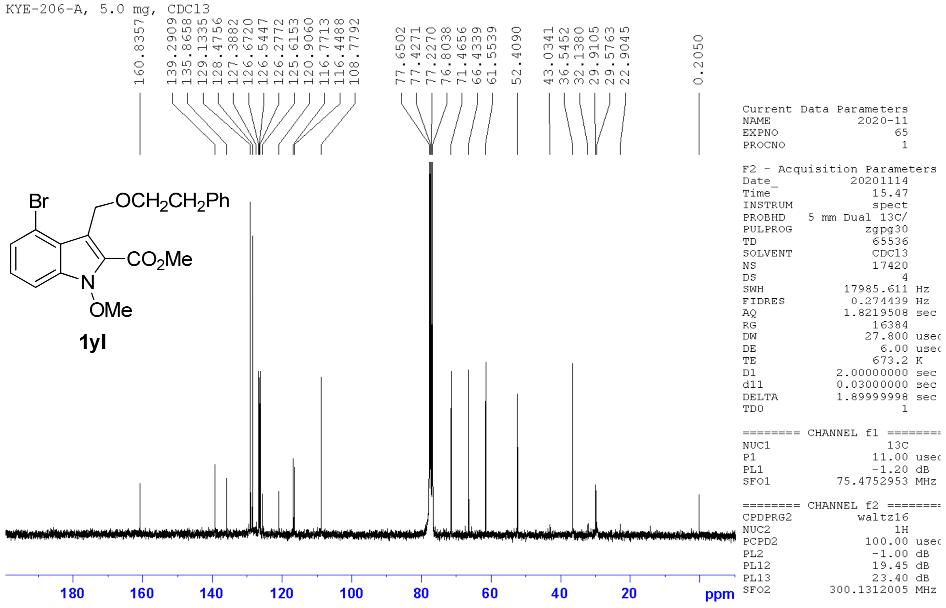

^{13}C NMR spectrum (75 MHz, CDCl$_3$) of compound 1yl

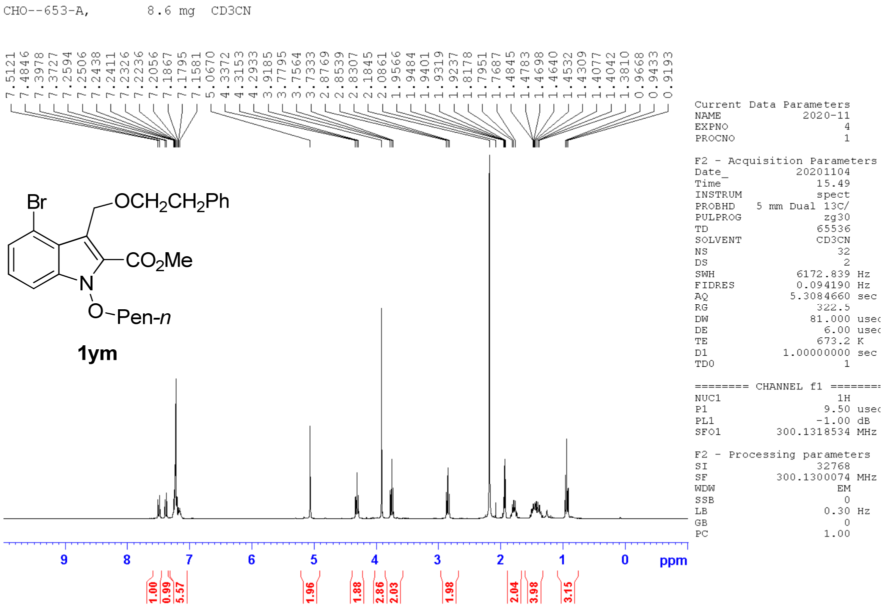

^{1}H NMR spectrum (300 MHz, CD_3CN) of compound 1ym

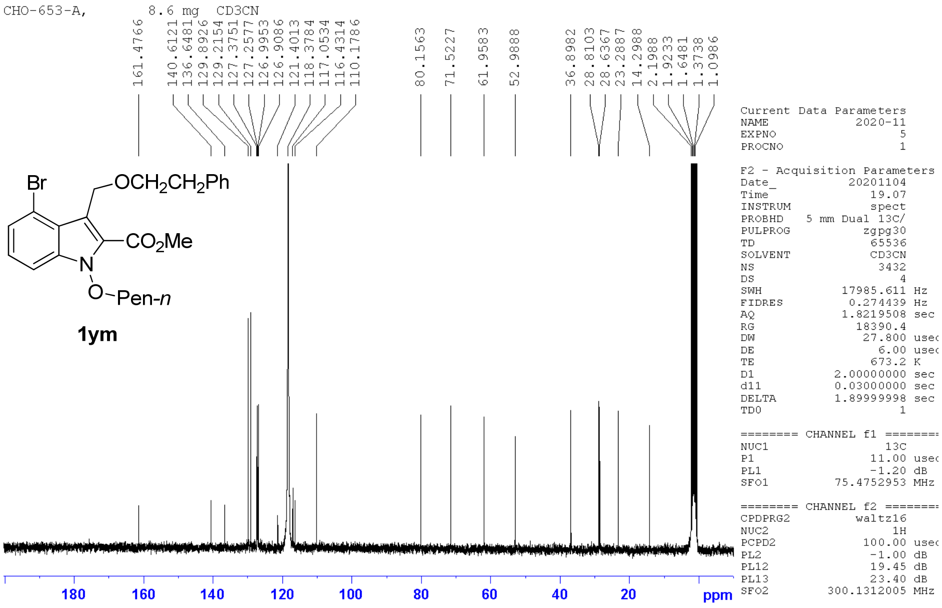

^{13}C NMR spectrum (75 MHz, CD_3CN) of compound 1ym

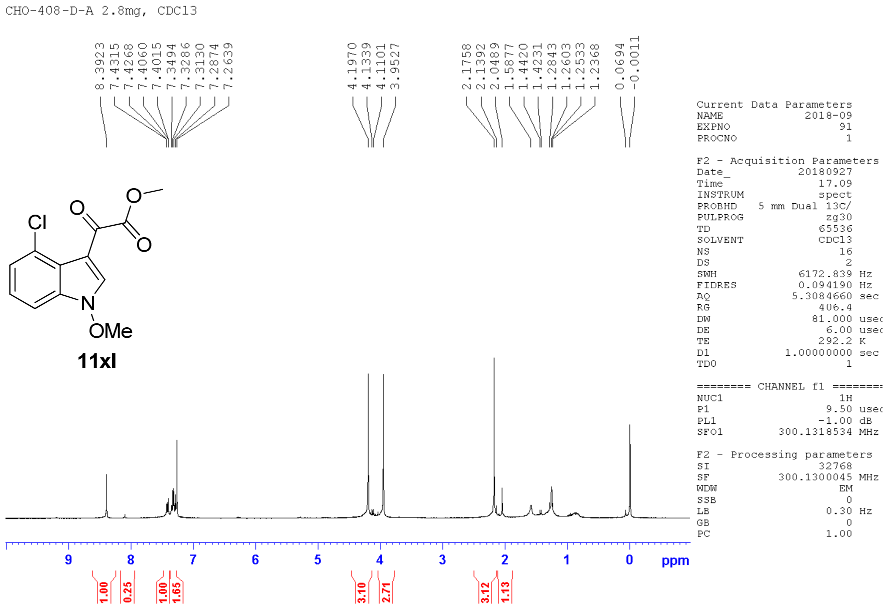

^{1}H NMR spectrum (300 MHz, $CDCl_3$) of compound 11xl

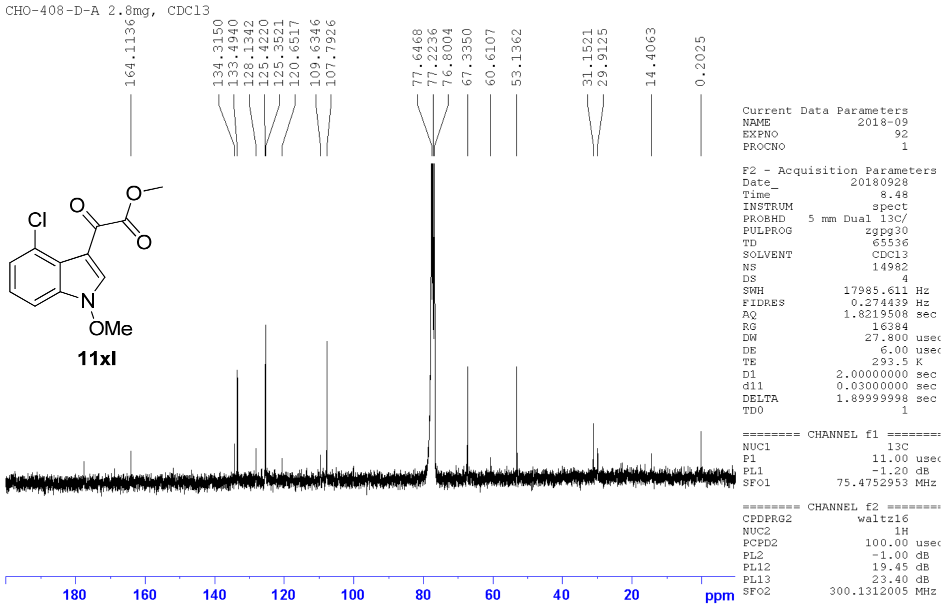

^{13}C NMR spectrum (75 MHz, $CDCl_3$) of compound 11xl

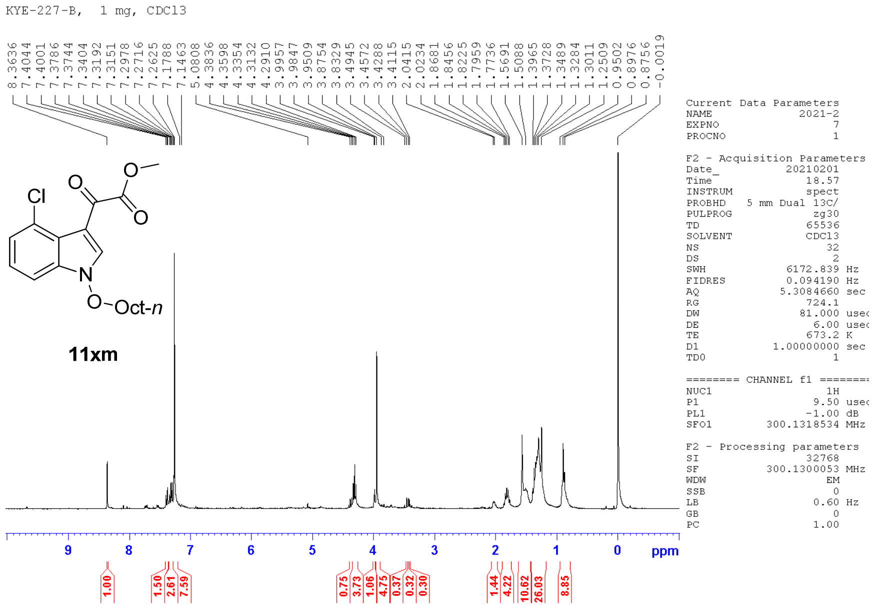

^{1}H NMR spectrum (300 MHz, $CDCl_3$) of compound 11xm

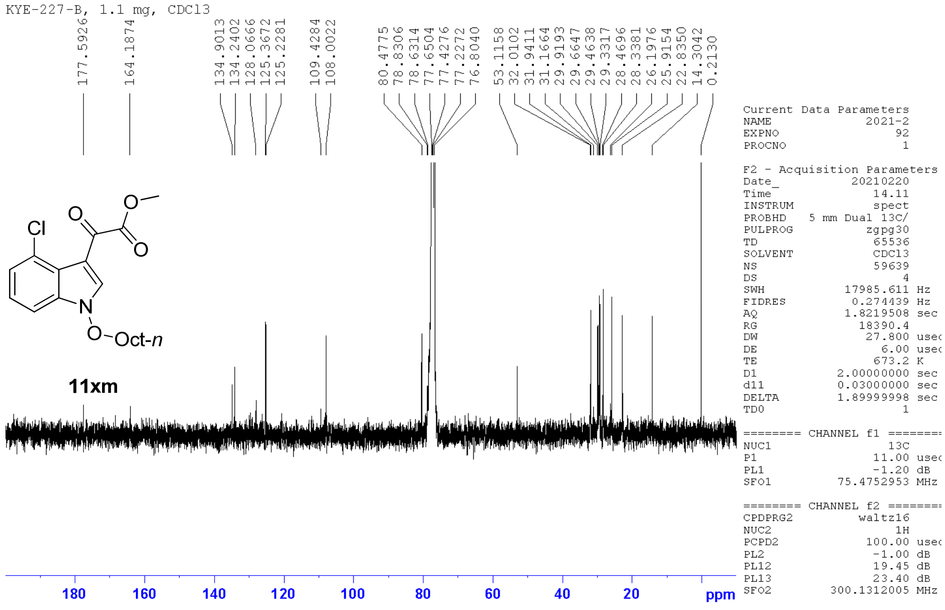

^{13}C NMR spectrum (75 MHz, $CDCl_3$) of compound 11xm
